# Bridging the gap: A systematic approach to integrating serum and plasma proteomic datasets for biomarker studies

**DOI:** 10.1016/j.jpba.2026.117421

**Published:** 2026-02-14

**Authors:** Coren Lahav, Nili Dahan, Michal Harel, Yehonatan Elon, Itamar Sela, Philipp E. Geyer, Marc A. Schneider, Thomas Muley, Antonella Bacchiocchi, Jennifer Marte, Charalampos S. Floudas, Ruth Halaban, Mario Sznol, Petros Christopoulos, James L. Gulley

**Affiliations:** a OncoHost Ltd., Binyamina, Israel; b ions.bio GmbH, Planegg, Germany; c Translational Lung Research Center, German Center for Lung Research (DZL), Heidelberg, Germany; d Translational Research Unit, Thoraxklinik, Heidelberg University, Heidelberg, Germany; e Department of Dermatology, Yale Cancer Center, Yale University School of Medicine, New Haven, CT, USA; f Center for Immuno-Oncology, Center for Cancer Research, National Cancer Institute, National Institutes of Health, Bethesda, MD, USA; g Department of Medicine, Division of Medical Oncology, Yale University School of Medicine, New Haven, CT, USA; h Department of Thoracic Oncology, Thoraxklinik, Heidelberg University Hospital, Heidelberg, Germany

**Keywords:** Proteomics, Serum, Plasma, Biomarker

## Abstract

Serum and plasma are widely used in proteomic biomarker discovery, but differences between their proteomes have hindered the integration of data from the two specimen types. Here, we describe a computational approach for bridging between serum and plasma proteomic measurements derived from the aptamer-based SomaScan assay. We aimed to enable cross-specimen data utilization in the context of the PROphet model designed to predict immunotherapy outcomes based on 388 plasma proteomic biomarkers. Proteomic profiling of 7289 proteins was performed on 177 matched serum-plasma sample pairs from cancer patients across three distinct cohorts. Remarkably, 91.6% of the proteins showed correlation (p-value < 0.05) between serum and plasma protein levels, highlighting the feasibility of serum-plasma bridging. Linear scaling factors derived from matched serum-plasma sample pairs were consistent across the three cohorts, suggesting that the scaling factors are generalizable. Notably, the PROphet model maintained its predictive power when applied to scaled serum proteomic measurements. Specifically, clinical benefit predictions and survival stratification based on scaled serum proteomic measurements were similar to those based on plasma proteomic measurements. Our study demonstrates the feasibility of generalizing plasma-based predictors to serum samples through appropriate bridging strategies, paving the way for integrating serum and plasma datasets.

## Graphical Abstract

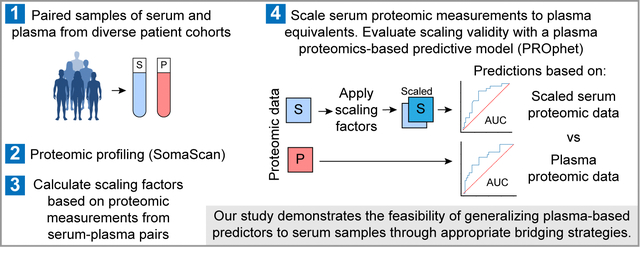

## 1. Introduction

Blood is the most common specimen used for routine clinical laboratory analysis [1]. Its liquid fraction, in the form of serum or plasma, contains a vast array of proteins and other molecules reflecting an individual’s physiological and pathological states. As such, these biofluids represent rich sources of potential biomarkers for risk prediction, diagnosis, prognosis, and therapeutic monitoring [2,3]. Proteomic profiling technologies fueling the discovery of blood-based proteomic biomarkers mainly include mass spectrometry [4], aptamer-based assays [5], and antibody-based proximity extension assays [6], all capable of quantifying hundreds to thousands of proteins in a single plasma or serum sample. Consequently, these technologies enable a broad exploration of the proteome. They also allow for the discovery of multi-protein biomarker signatures that potentially offer higher discriminatory power than single-protein biomarkers by better capturing the complexity of biological phenomena. Indeed, large-scale data mining efforts have resulted in the discovery of novel proteomic biomarker signatures across various diseases [7–11].

While both serum and plasma have been used extensively in proteomic studies, the proteomes of the two specimen types cannot be directly compared [12]. Plasma is prepared from whole blood by adding anticoagulants such as EDTA, heparin, or citrate, followed by centrifugation to remove the cellular material. Serum, on the other hand, is prepared by allowing the blood to clot naturally. The fibrin clot and cellular material are then removed by centrifugation, resulting in a fluid depleted of fibrinogen and other factors that interact with the clot [13]. Notably, the clotting process is associated with the secretion of specific proteins by platelets, red blood cells, and white blood cells, further contributing to the differences between serum and plasma proteomes [12]. Given these proteomic differences, biomarker studies are generally restricted to either one of the two specimen types. Cross-specimen data utilization could expand the biomarker research scope by enabling multi-cohort studies regardless of specimen type.

Some studies have explored the feasibility of cross-specimen proteomic biomarker signatures. For example, Espinosa et al. developed models for predicting gestational age based on proteomic signatures in maternal serum or plasma. They showed that predictive information in serum is preserved in plasma, whereas predictive information in plasma is not fully preserved in serum [14]. In another study analyzing 358 proteins relevant to obesity-related diseases, most proteins displayed similar concentrations in serum and plasma. However, some key biomarkers within the protein set showed poor serum-plasma correlations [15]. While these studies demonstrate some degree of compatibility between the two matrices, they also highlight the need to address serum-plasma differences that may compromise cross-specimen data utilization. Methodologies for bridging between serum and plasma proteomic data are scarce; to date, there is only one published study describing serum-plasma transformation factors for ~600 Olink-quantified proteins [16].

Here, we describe a computational approach for bridging between serum and plasma proteomic datasets derived from the aptamer-based SomaScan assay. Our approach enables cross-specimen data utilization in the context of the previously described plasma proteomics-based PROphet model [8]. The PROphet model is designed to predict immunotherapy outcomes in patients with non-small cell lung cancer (NSCLC) based on 388 pretreatment plasma proteomic biomarkers. This study investigates its performance when scaled serum proteomic measurements are used as input. The bridging process is based on linear scaling factors computed from matched serum-plasma proteomic dataset pairs that have passed compatibility and quality checks. We demonstrate that the PROphet model maintains its predictive power when applied to scaled serum proteomic measurements, confirming the validity of the scaling process and suggesting the preservation of underlying biological information within the scaled dataset. Our systematic approach may be applied in other contexts, potentially enabling the integration of diverse datasets for biomarker research.

## 2. Materials and methods

### 2.1. Patient cohorts

The study is based on multiple patient cohorts (Supplementary Table S1A). All participants provided written informed consent. All clinical sites received approval from their local Institutional Review Board (Supplementary Table S1B).

Matched plasma and serum samples, totaling 184 pairs, were sourced from three distinct cohorts, designated cohort A, cohort B, and cohort C. Cohort A comprised 55 NSCLC patients treated with PD-1/PD-L1 inhibitor-based therapies at Thoraxklinik, Heidelberg University Hospital, Heidelberg, Germany (NCT04056247). In this case, samples were collected before treatment. Samples were provided by Lung Biobank Heidelberg, a member of the BiomaterialBank Heidelberg (BMBH) and the platform biobanking of the German Center for Lung Research (DZL). Cohort B was comprised of 44 patients with HPV-related malignancies treated at the National Cancer Institute, Bethesda, MD, USA (NCT03427411). A total of 82 sample pairs were collected, of which 43 and 39 were collected pre- and on-treatment, respectively. Cohort C comprised 47 metastatic melanoma patients treated at Smilow Cancer Hospital-Yale New Haven Health, New Haven, CT, USA. In this case, samples were collected before treatment. Patient characteristics are provided in Supplementary Tables S2, S3 and S4.

Unpaired samples from 636 subjects (533 plasma samples and 103 serum samples) served as external reference samples. Plasma samples were sourced from 445 NSCLC patients and 88 melanoma patients participating in the PROPHETIC clinical trial (NCT04056247) across 20 centers in Israel, Germany, the United Kingdom, and the United States. Serum samples were sourced from 59 patients with metastatic melanoma treated at Smilow Cancer Hospital-Yale New Haven Health, New Haven, CT, USA and 44 patients with HPV-related malignancies treated at the National Cancer Institute, Bethesda, MD, USA (NCT04287868). Two plasma reference subsets were used for the sample contamination analysis. Specifically, samples from the 445 NSCLC patients were split into two subsets of 409 samples prepared according to standard protocol and 36 samples prepared using high-speed centrifugation. Specimen preparation protocols are detailed below. In all cases, samples were collected before treatment. There was no overlap between the reference cohorts and cohorts A, B, and C.

### 2.2. Sample preparation

For plasma preparation, whole blood was collected by venipuncture and placed into EDTA-anticoagulant tubes. Plasma was isolated by centrifugation, either by a single spin at 2000 g for 10 min (cohort A) or by two sequential 10-minute spins at 495 g followed by 1170 g (cohort B), or 800 g followed by 450 g (cohort C). Reference plasma samples were prepared by centrifugation at 1100 g – 2000 g (standard protocol) or by high-speed centrifugation at 16000 g. For serum preparation, whole blood was collected into serum-separating tubes. Blood was allowed to clot at room temperature for 30 min. Serum was isolated by centrifugation at 800–2000 g for 10 min. All samples were frozen at −80°C and shipped to the analysis laboratory. Further details on specimen preparation protocols per cohort are provided in Supplementary Tables S5 and S6.

### 2.3. Proteomic measurements

Proteomic profiling of all plasma and serum samples was performed using the SomaScan Assay v4.1 at OncoHost’s CLIA-certified, CAP-accredited laboratory (Cary, NC). The assay is based on Slow Off-Rate Modified Aptamers (SOMAmers) - engineered single-stranded oligonucleotides designed to fold into unique three-dimensional configurations that bind target proteins with high specificity and affinity [17–19]. The assay measures 7596 protein targets of which 7289 are human protein targets. Assay results are reported in relative fluorescence units (RFU). The analytical validity of this assay, including inter- and intra-plate precision and accuracy, was previously reported in the context of the PROphet model [20]. We adhered to the standardized quality control protocols established for the SomaScan platform [5] to ensure data quality and consistency.

### 2.4. Sample exclusion

**Fig. 1.**
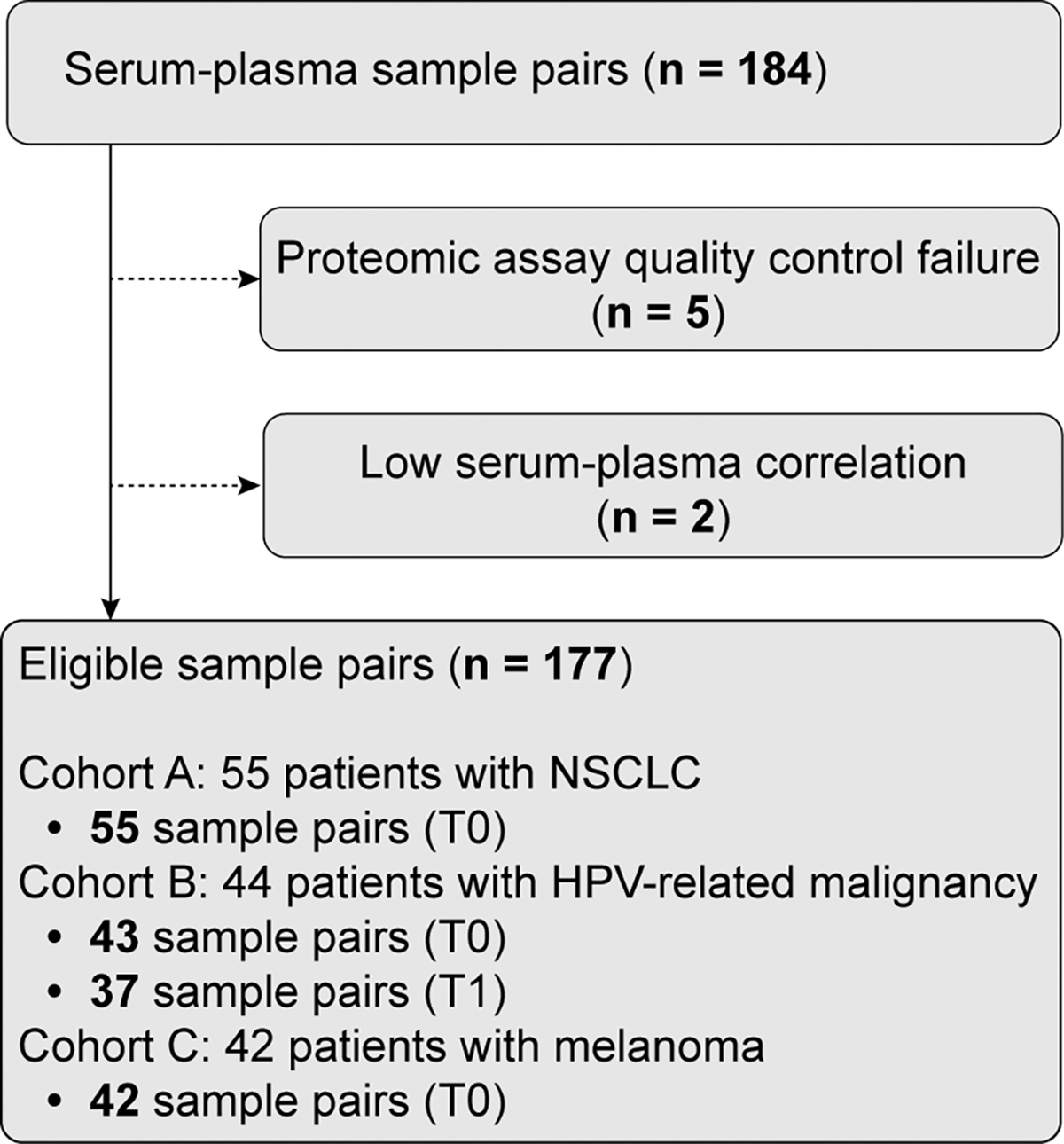
Study cohort overview. Paired serum and plasma samples were analyzed (n = 184 pairs; n = 177 pairs after exclusions). Reasons for exclusions are indicated. Samples were sourced from three cancer patient cohorts before treatment (T0) and sometimes during treatment (T1). NSCLC, non-small cell lung cancer; HPV, human papillomavirus.

Of the 184 pairs of serum and plasma samples, seven were excluded, resulting in 177 pairs eligible for analysis (Fig. 1). Specifically, five sample pairs were excluded based on the failure of one of the samples within the pair to meet the SomaScan quality control criteria (i.e., normalization scale factors within the range of 0.4–2.5, as previously described [21,22]). Two sample pairs were excluded due to pairing failure manifested by unusually low correlation between paired samples (Supplementary Figure S1; see outlier definition).

### 2.5. Outlier definition

We employed the 1.5 interquartile range (IQR) method for defining outliers across multiple analyses [23]. Specifically, low and high outliers were defined using Equation 1 and 2, respectively.

Equation 1: Lower threshold for outliers

Q1-1.5•(Q3-Q1)

Equation 2: Upper threshold for outliers

Q3+1.5•(Q3-Q1)

where Q1 and Q3 are the first and third quartiles of the distribution, respectively.

The method was applied in three contexts. (i) Sample exclusion: protein levels were normalized using z-scores (separately for serum and plasma proteomic profiles across the three paired cohorts). Serum-plasma correlations were calculated per paired sample based on the 388 PROphet biomarkers. IQR-based outlier correlation thresholds were calculated across all sample pairs, and sample pairs were excluded if their correlation fell below the threshold for low outliers; (ii) Plasma-serum protein expression ratios: the outlier approach was used for defining high plasma (upper outliers) and high serum (lower outliers) proteins based on ratios calculated over the three patient cohorts; (iii) Concordance between internal and external datasets: Outliers were identified based on the distribution of differences in median values between paired and reference samples. Outlier median differences were defined separately for serum and plasma.

### 2.6. Data normalization

Normalization was used in the following analyses: characterization of the differences between serum and plasma datasets; sample contamination assessment. Datasets were normalized separately for each specimen type (serum and plasma) across all patient cohorts using iterative scaling which combines z-score standardization with outlier handling. This method ensures robust normalization of skewed distributions. It works as follows: (i) Perform z-score standardization. (ii) Identify outliers as values exceeding 4 standard deviations from the mean. (iii) Temporarily exclude outliers and repeat steps 1–2 until no new outliers are identified. (iv) Impute all outlier values; those above the mean are set to + 4 standard deviations, and those below are set to −4 standard deviations of the modified distribution.

To validate that normalization findings were not attributable to outlier handling, differences between serum and plasma datasets were also assessed using robust scaling. Robust scaling preserves extreme values by scaling each protein via subtracting the median and dividing by the IQR.

### 2.7. Enrichment analyses

Gene Ontology (GO), Kyoto Encyclopedia of Genes and Genomes (KEGG) and Keywords (Uniprot) databases were used for functional enrichment analysis performed on proteins grouped by serum-plasma correlation strength. To evaluate sample quality, enrichment analyses were performed with reference to four quality panels defined by Geyer et al., 2019 [24]: platelet contamination, erythrocyte contamination, and two categories of coagulation markers (i.e., those increased in plasma relative to serum and those increased in serum relative to plasma). All enrichment analyses were performed using the Fisher exact test with Benjamini-Hochberg false discovery rate (FDR) < 0.1.

### 2.8. Serum measurement scaling

Statistical evaluation demonstrated that the majority of proteins exhibited linear relationships between serum and plasma measurements, with apparent non-linearity predominantly driven by outliers. Thus, a linear model was chosen as the most suitable approach for serum-to-plasma scaling.

Serum-to-plasma scaling factors were calculated from serum-plasma pairs using linear regression, minimizing the sum of squares of the residuals. No regularization was applied. This was done at the single-protein level, such that scaling is based on two parameters per protein: regression slope and intercept. Three scaling factor sets were generated: set 1 from cohort A pairs (using k-fold cross-validation), set 2 from cohort B pairs, and set 3 from cohort C pairs. A fourth set was generated as a negative control from cohort A mismatched pairs created by random shuffling of plasma samples with respect to serum samples. Serum measurements from the cohort A dataset were then scaled to their plasma equivalents by applying Equation 3.

Equation 3: Serum measurement scaling

$${log}_{2}(RFU{)}_{\mathrm{Scaled}}={log}_{2}(RFU{)}_{\mathrm{Raw}}∙Slope\;+\;Intercept$$

### 2.9. The PROphet model

The PROphet model, designed to predict PD-1/PD-L1 inhibitor treatment outcomes in patients with NSCLC, was previously trained on a set of 228 patients and evaluated on an independent set of 272 patients. It is based on 388 pretreatment plasma proteomic biomarkers. The PROphet model computes the probability of clinical benefit (CB), defined as progression-free survival at 12 months, and categorizes patients as either PROphet-POSITIVE or -NEGATIVE based on a predefined CB probability threshold [8]. Model development and validation are described in detail by Christopoulos et al. [8] and Yellin et al. [20]. Cohort A of the current study is a subset of the cohort used previously for model validation. Predictive performance was evaluated using the Receiver Operating Characteristic Area Under the Curve (ROC AUC) and by assessing overall survival in patients classified as PROphet-POSITIVE or -NEGATIVE.

### 2.10. Development and validation of other predictive models

Models for predicting patient sex and two NSCLC co-morbidities, namely hypertension and chronic obstructive pulmonary disease (COPD), were trained using the same methodology and dataset used for PROphet model development based on plasma samples from 228 NSCLC patients participating in the PROPHETIC clinical trial (NCT04056247) [8]. Cohort A of the current study served as the independent validation cohort. Trait frequency distributions in the development and validation sets are provided in Supplementary Table S7.

### 2.11. Statistical analysis

Data analysis was performed using Python v3.12.5. Plasma-to-serum ratios were calculated based on raw RFU values. All other analyses were performed on log_2_-transformed RFU values. The dataset was complete with no missing values; therefore, no imputation or handling of missing data was required. For inter-sample correlation analysis, log_2_-transformed measurements were z-score normalized per protein. Principal component analysis (PCA), hierarchical clustering and linear regression were performed using the scikit-learn Python package v1.5.2. The Ward method was used for hierarchical clustering. The lifelines Python package v0.30.0 was used for Kaplan-Meier curve plotting, hazard ratio calculation, and log-rank test. Perseus v1.6.2.3 software was used for enrichment analysis [25].

## 3. Results and discussion

### 3.1. Quantifying the differences between serum and plasma proteomes

**Fig. 2.**
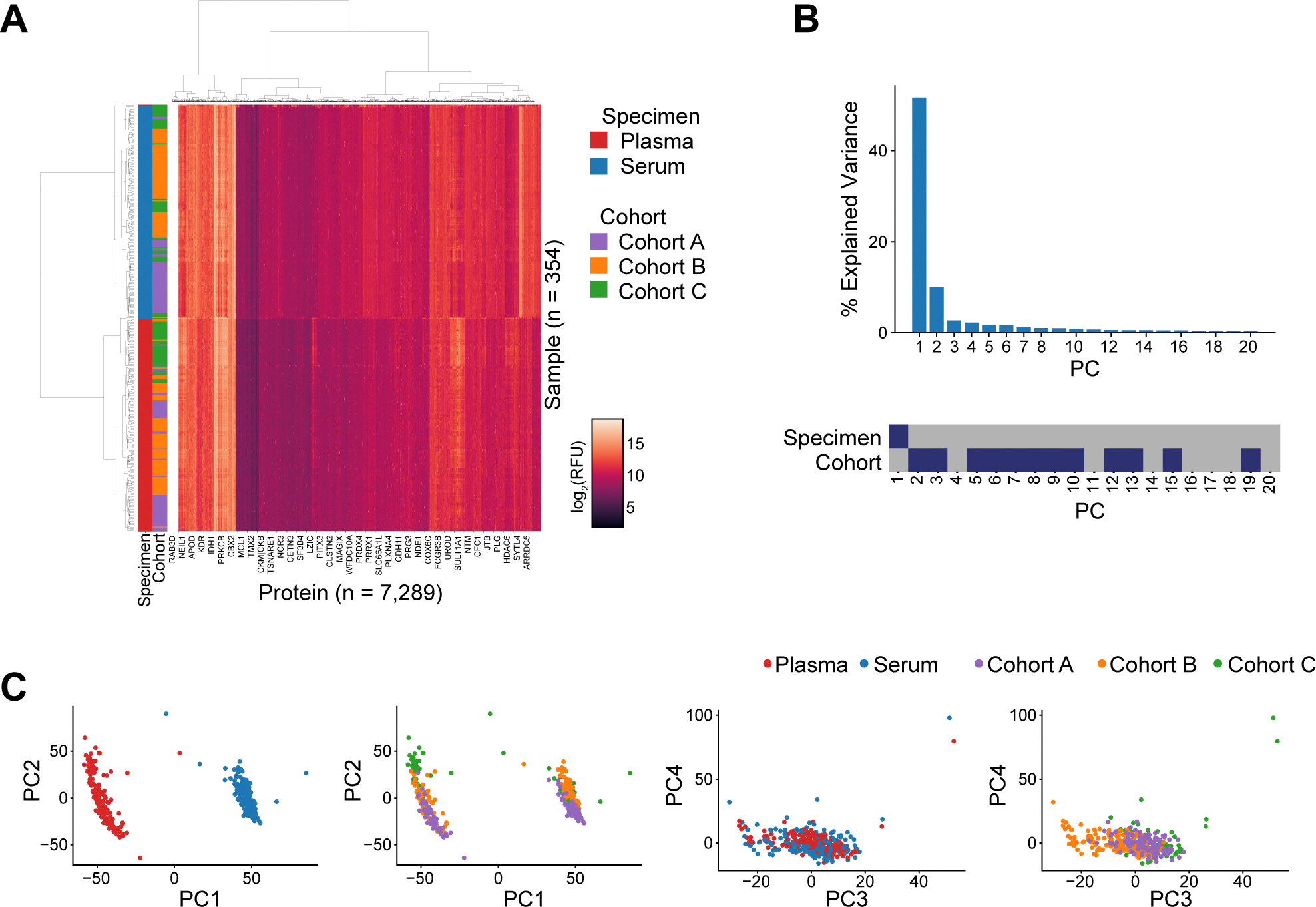
Differences between plasma and serum proteomes. (A) Hierarchical clustering of log-transformed protein measurements of plasma and serum samples from three cohorts. (B) Principal component analysis. The bar graph shows the percentage of explained variance by the top 20 principal components (PCs) (top). Association of the top 20 PCs with specimen type and cohort (bottom). Blue, p < 0.05; gray, not significant. (C) Scatterplots of PC values for the top four PCs showing association with specimen type and cohort.

To develop a computational approach for bridging between serum and plasma proteomic data, we first compared serum and plasma proteomes on a broad level. To this end, we assembled 184 matched pairs of serum and plasma samples from cancer patients across three distinct cohorts referred to as cohort A (patients with NSCLC), cohort B (patients with HPV-related malignancies), and cohort C (patients with melanoma). Each cohort originated from a different clinical site with study specific sample collection protocols (Supplementary Tables S1, S5 and S6). Aptamer-based SomaScan proteomic profiling measuring 7289 human protein targets was performed for all samples. Seven sample pairs were excluded based on quality control (QC) criteria, as described in Materials and Methods, resulting in 177 eligible pairs for further analysis (Fig. 1). Hierarchical clustering of the proteomic measurements revealed a clear separation between serum and plasma samples, forming two primary clusters with only one plasma sample falling within the serum cluster (Fig. 2A). Within each cluster, samples from the same cohort tended to group together, albeit with imperfect separation between cohorts. These cohort-based subgroups likely reflect a combination of preanalytical variability (due to differences in sample preparation protocols per cohort) and biological variability (as each cohort represents a different cancer type). However, the imperfect separation of cohorts suggests these differences are less pronounced than the serum-plasma distinction. This is further supported by the dendrogram structure, where the height of the branches separating serum and plasma clusters is substantially greater than those distinguishing between sub-clusters, quantitatively demonstrating that the specimen type differences dominate over cohort-specific variations.

To further investigate the sources of variability in our dataset spanning 7289 proteins, we applied principal component analysis (PCA) to the protein measurements. The first principal component (PC), explaining 46.9 % of the total variability, completely separated serum and plasma samples, confirming that specimen type is the dominant source of variation in our data (Fig. 2B-C). Of the other top 20 PCs, none were associated with specimen type, while 13 were significantly associated with cohort (Fig. 2B, bottom panel). Furthermore, after iterative scaling (z-normalization combined with an outlier detection approach, see Materials and Methods), none of the top 20 PCs were significantly associated with specimen type, removing most of the variability introduced by serum-plasma differences (Supplementary Figure S2A-B). To confirm that outlier exclusion inherent to iterative scaling did not mask extreme but true biological phenomena, we also assessed the effect of robust scaling. In this case, only PC6 and PC11 (cumulatively explaining 3 % of the total variability, rather than the original 46.9 %) were significantly associated with specimen type (Supplementary Figure S2C-D).

Overall, these findings indicate that multiple properties of the dataset are associated with biological and/or pre-analytical properties rather than with specimen type.

### 3.2. Characterizing the correlations between serum and plasma proteomes

**Fig. 3.**
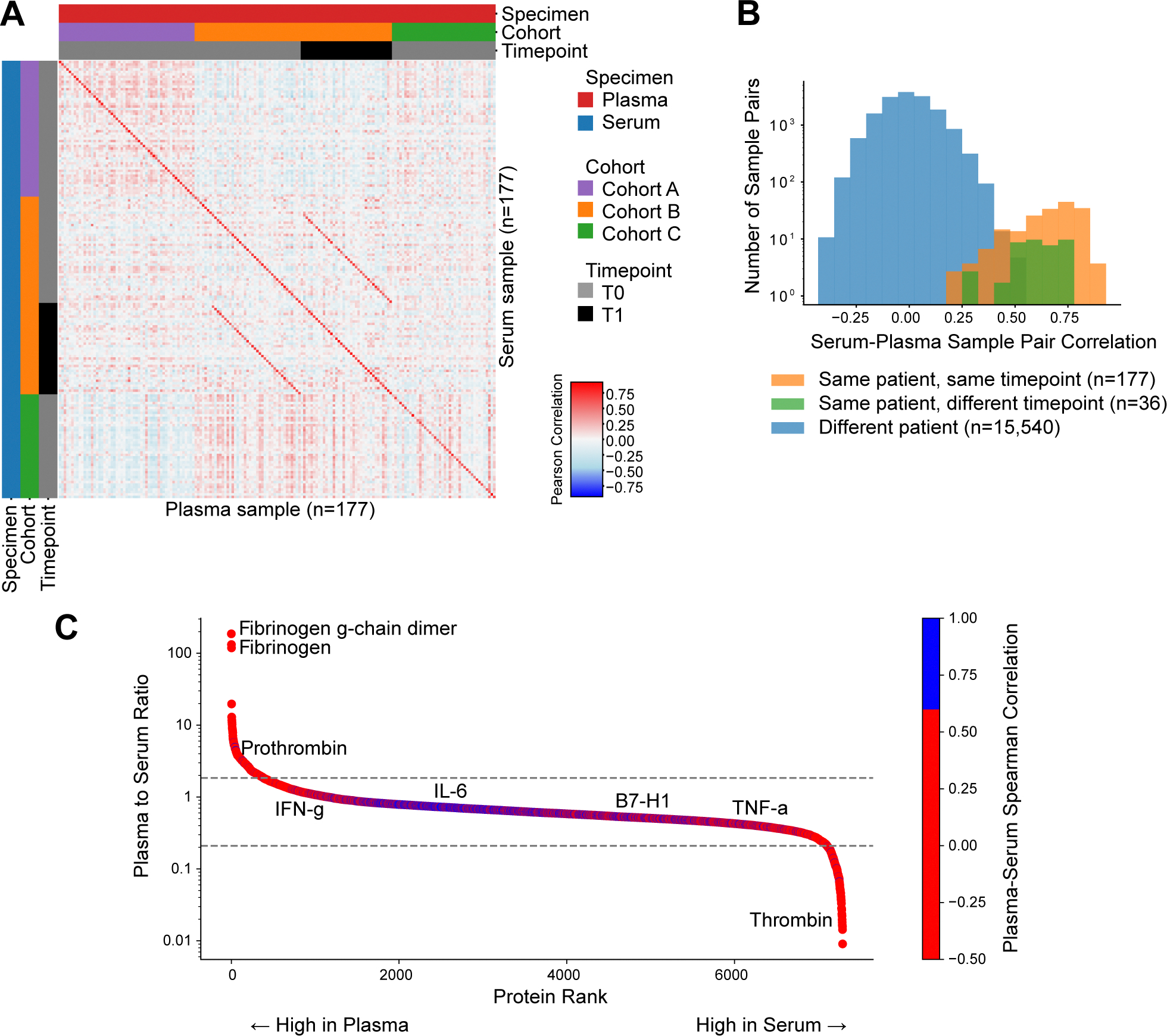
Inter-sample correlations between plasma and serum proteomes. (A) Inter-sample plasma-serum protein expression correlations. Paired samples are located on the main diagonal. Pretreatment (T0) and on-treatment (T1) samples collected from the same patient were sorted to generate a secondary diagonal (which is mirrored by pairing symmetry). (B) Histogram of correlations between serum and plasma datasets. Pearson correlation between serum-plasma pairs (orange), samples collected from the same patient at different timepoints (green), and all possible unpaired, independent sample combinations (blue). (C) Association between median plasma-to-serum ratio and plasma-serum protein correlation. X-axis: proteins are sorted by plasma-to-serum ratio. Y-axis: median plasma-to-serum ratio (log scale). Dashed lines indicate thresholds for high plasma-to-serum (upper) and high serum-to-plasma (lower) sets of proteins used in the enrichment analysis. Analyses are based on 7289 protein analytes across cohorts A, B, and C.

We next examined the correlations between serum and plasma proteomes. In an inter-sample correlation matrix of serum and plasma samples, two distinct patterns of high correlation were apparent (Fig. 3A). The main diagonal represents the correlation between paired serum and plasma samples from the same patient and time point, demonstrating a strong similarity despite the difference in specimen type. This finding agrees with previous analyses that report stability between sample pairs of different specimens [26,27]. Remarkably, the secondary diagonal shows high correlations between samples from the same patient collected at different time points (pre-treatment and on-treatment), regardless of whether they are serum or plasma. Importantly, these within-patient correlations were substantially higher than those between patients within the same cohort (Fig. 3B, Kruskal-Wallis p-value < 0.0001). This observation demonstrates that patient-specific biological signals are preserved across proteomes of both serum and plasma matrices and remain stable in samples collected weeks apart. These findings suggest that while specimen type accounts for a large portion of overall variability (as demonstrated in Fig. 2), it does not obscure underlying biological information relevant to biomarker research.

To further characterize the agreement between serum and plasma measurements, we compared Pearson and Spearman correlations per measured protein across the three cohorts. In most cases, Pearson correlations were higher than Spearman correlations (Supplementary Figure S3A). This was frequently due to a pattern where most protein values clustered near the limit of detection with poor measurement precision, while a small number of outlier values far from the limit of detection showed strong agreement between serum and plasma. Since Pearson correlation is sensitive to extreme values, these rare but concordant outliers disproportionately inflated Pearson correlations, as demonstrated in plasma-serum scatterplots for selected proteins (Supplementary Figure S3B-C; e.g., TNF-a, DAPK1, SMAD1). Indeed, there was an association between the distance of measured protein values from the limit of detection and the degree to which Pearson correlations exceeded Spearman correlations (Supplementary Figure S4, Spearman r = −0.61, p < 0.0001). While these outliers represent biologically relevant measurements, their rarity implies they should not dominate the assessment of overall serum-plasma agreement. Given this, we used the Spearman method to evaluate plasma-to-serum agreement at the protein level.

Of the 7289 examined proteins, 6680 (91.6 %) displayed serum-plasma correlation p-values below 0.05 (Spearman’s r, unadjusted, Supplementary Table S8), far exceeding the 5 % of proteins that would be expected by chance alone. The remarkably high percentage of correlated proteins highlights the feasibility of serum-plasma bridging.

A closer examination of individual protein behaviors revealed a more nuanced picture. Fig. 3C presents the plasma-to-serum ratio for 7289 human protein analytes, ranked and sorted along the x-axis. Most proteins exhibited plasma-to-serum ratios close to 1, indicating similar concentration scales in both matrices. However, two smaller subgroups of proteins were evident as shoulders deviating from the central group. At the extremes, we observed proteins with ratios exceeding 100 (i.e., more than 100 times higher in plasma than serum) and approaching 0.01 (i.e., nearly 100 times higher in serum than plasma). Some of these proteins are known to be related to the coagulation process. For example, fibrinogen was among the most depleted proteins in serum, and thrombin was among those most depleted in plasma. Furthermore, enrichment analyses of the proteins at either end of the distribution (above and below the upper and lower thresholds, respectively) confirmed established patterns of coagulation markers (Supplementary Table S9). Specifically, proteins with high plasma-to-serum ratios matched known plasma-retained coagulation factors, while proteins with low ratios showed a trend toward serum-accumulated coagulation factors [24]. Additionally, proteins with high plasma-to-serum ratios were enriched with platelet and erythrocyte contamination markers, indicating higher contamination levels in the plasma samples.

Examining plasma-serum Spearman correlation coefficients per protein along the distribution revealed further insights. Specifically, the central part of the distribution (i.e., where proteins displayed plasma-to-serum ratios close to 1) was predominantly composed of proteins with a high correlation between serum and plasma measurements. In contrast, the proteins beyond the thresholds displayed lower correlation (Fig. 3C). This pattern demonstrates that a significant source of disagreement between plasma and serum measurements is associated with a minority of proteins. Enrichment analysis of proteins categorized by serum-plasma correlation strength revealed some interesting insights (Supplementary Figure S5, Supplementary Table S10). Proteins showing strong serum–plasma correlation were enriched with extracellular and secreted proteins, likely because they are stably present in the circulation and are minimally affected by clotting or sample handling. Consistently, proteins involved in glycosylation and glycan biology were also enriched among highly correlated proteins, possibly because glycosylation enhances protein stability and prolongs circulating half-life, leading to more consistent quantification across matrices. In contrast, proteins showing poor serum–plasma correlation were predominantly cell-associated or intracellular, including those related to cell cycle, RNA processing, cytoskeletal organization, and protein homeostasis or turnover. These proteins are more susceptible to cellular activation, coagulation processes, or lysis during sample preparation, which may explain variable detection between serum and plasma.

### 3.3. Scaling factors for integrating serum and plasma proteomic data

**Fig. 4.**
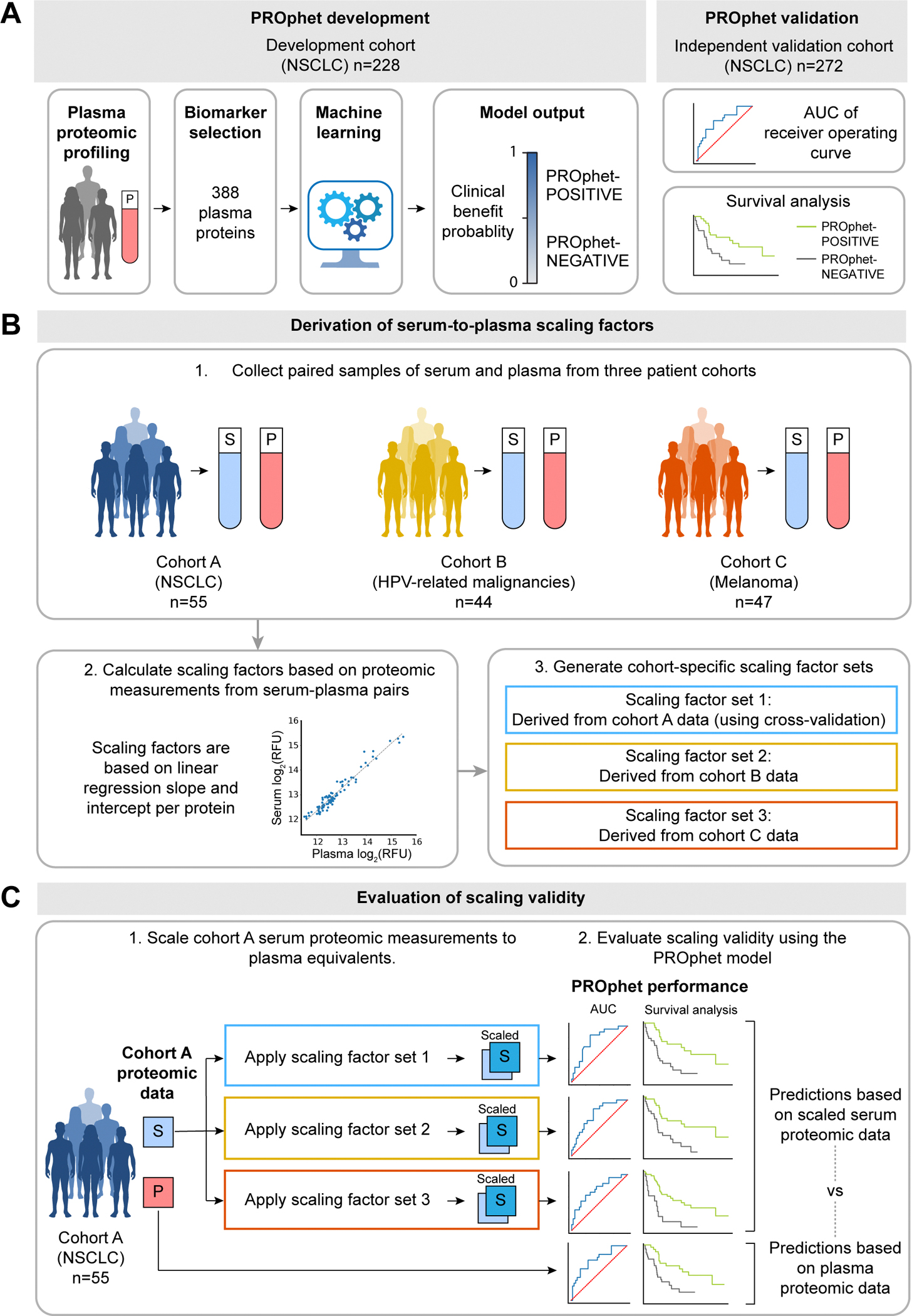
Methodology for serum-to-plasma scaling of proteomic measurements. (A) PROphet model development and independent validation were previously reported [8] and are schematically presented in the figure. (B) Derivation of serum-to-plasma scaling factors. Paired serum–plasma samples from three independent cohorts were used to compute protein-specific linear scaling factors. (C) Evaluation of scaling validity in the context of the PROphet model. Cohort A was used as the scaling validation cohort. Serum samples from cohort A were scaled using each factor set (derived from cohorts A, B, C). PROphet predictions based on scaled serum proteomic measurements were compared to those from plasma. AUC, area under the curve; P, plasma; S, serum.

In this study, we specifically aimed to scale serum proteomic measurements to their plasma equivalents, allowing serum samples to be used in the context of the plasma proteomics-based PROphet model. The PROphet model relies on 388 pretreatment plasma proteomic biomarkers to predict PD-1/PD-L1 inhibitor treatment outcomes in patients with NSCLC [8,20]. Specifically, it computes the probability of achieving CB (defined as progression-free survival at 12 months) from treatment. It also stratifies patients based on survival outcomes by providing a PROphet-POSITIVE versus PROphet-NEGATIVE binary output, as demonstrated by a validation study showing significantly longer survival in patients classified as PROphet-POSITIVE compared to PROphet-NEGATIVE [8]. A brief description of the PROphet model is provided in Fig. 4A. Here, we designed an approach to explore the robustness and generalizability of serum-to-plasma scaling in the context of PROphet (Fig. 4B-C). The approach involves generating scaling factor sets from matched pairs of serum and plasma samples from diverse cohorts (i.e., cohorts A, B and C), allowing us to assess reproducibility across different clinical and pre-analytical conditions. The three scaling factor sets are then used to scale serum proteomic measurements to plasma equivalents. Scaling validity is assessed by applying the PROphet model to scaled serum data and comparing scaled serum-based predictions to plasma-based predictions. In this case, cohort A serves as the scaling validation cohort; it is suitable for evaluation by PROphet as it consists of patients with NSCLC treated with PD-1/PD-L1 inhibitors. Note that cohort A scaling factors are generated using a cross-validation approach allowing them to be applied to the same cohort from which they are generated. The cross-validation approach is not needed in the case of the independent cohorts B and C.

Before calculating scaling factors from serum-plasma pairs derived from cohorts A, B and C, we analyzed the datasets to identify and address issues that might introduce bias or compromise model performance. We focused on the following four aspects:

- Assuming that proteins displaying a low correlation between serum and plasma measurements will not scale well, we reasoned that such proteins should be filtered out from the predictive model. However, we found that the set of 388 biomarkers used by the PROphet model was already enriched with proteins displaying high correlations between serum and plasma measurements (Supplementary Figure S6, Mann-Whitney p-value < 10^−60^). This is due to various filtering steps applied during model development (i.e., pre-analytical filtering steps that selected proteins with the highest stability across different plasma separation methods followed by feature selection) [8], as shown in Supplementary Figure S6. Based on this, we opted to work with the complete set of 388 proteomic biomarkers without further filtering.
- We identified sample pairs displaying a low correlation between serum and plasma measurements and excluded them from the set to be used for scaling factor calculation (Supplementary Figure S1; see Materials and Methods for details).
- To evaluate sample quality, we tested for platelet and erythrocyte contamination using previously described proteomic marker panels [24]. Measurements of the platelet and erythrocyte proteomic markers in plasma and serum samples from cohorts A, B, and C were normalized via iterative scaling against reference plasma samples from an external, independent cohort (described in Materials and Methods). We also included 36 plasma samples prepared using high-speed centrifugation (16000 g), representing a positive control for minimal platelet contamination [28]. Hierarchical clustering of the normalized values revealed that platelet and erythrocyte marker levels were extremely low in the positive control samples, as expected. Notably, the platelet and erythrocyte markers tended to be elevated in cohort C plasma samples compared to cohorts A and B (Supplementary Figure S7A and S7B). Furthermore, platelet and erythrocyte contamination scores (calculated by averaging normalized measurements per patient) were higher in cohort C plasma samples, while serum samples across all cohorts displayed minimal contamination (Supplementary Figure S7C and S7D). Thus, cohort C plasma samples were flagged as a possible source of bias.
- We posited that scaling factors calculated from paired serum-plasma samples will be reliable and applicable to datasets from other cohorts if the paired samples are representative of a broader population. To verify whether our paired samples fulfilled this criterion, we compared plasma or serum datasets from cohorts A, B, and C against those from external reference cohorts. For simplicity, we refer to these analyses as: (i) ‘plasma-plasma’ analysis, where cohort A, B, or C plasma datasets were compared to the external reference plasma dataset; (ii) ‘serum-serum’ analysis, where cohort A, B, or C serum datasets were compared to the external reference serum dataset. This was done by assessing correlations between the median values per protein in the compared sets. The analyses were performed for the entire set of 7289 proteins and for the protein set composed only of PROphet biomarkers (388 proteins). In the plasma-plasma analysis, plasma protein measurements from cohorts A and B correlated with those from the external plasma cohort, indicating that cohort A and B plasma samples represent a broader population (Supplementary Figure S8A-D; Supplementary Table S11). In contrast, cohort C plasma samples showed poor correlation to those from the external cohort when analyzing the entire set of 7289 proteins, with a subset of proteins forming a large tail deviating from the diagonal (Supplementary Figure S8A-B). The tail was less apparent when analyzing the smaller set composed of 388 PROphet biomarkers. However, the overall deviation from the diagonal was still larger than that displayed by cohorts A and B (Supplementary Figure S8C-D). Overall, these findings suggest that cohort C plasma samples contain variability that might hamper the success of the serum-plasma integration process. The variability likely stems from pre-analytical factors, consistent with the higher platelet and erythrocyte contamination detected in these samples (Supplementary Figure S7). Indeed, proteins within the distribution tail per cohort were significantly enriched with platelet and erythrocyte contamination markers, while cohort C displayed the largest tail (Supplementary Table S12). The serum-serum analysis revealed high correlations between serum samples from all tested cohorts and the external serum cohort (Supplementary Figure S9). Thus, all cohorts passed the quality criteria for serum samples, while only cohorts A and B passed for plasma samples.

Next, we calculated serum-to-plasma scaling factors using linear regression at the single protein level per cohort. Since the final goal was to apply the scaling factors to cohort A serum measurements, they were calculated from cohort A serum-plasma pairs using k-fold cross-validation or from the independent cohort B or C serum-plasma pairs. As a negative control, we used mismatched serum-plasma pairs created by randomly shuffling cohort A plasma samples with respect to serum samples. An analysis of the regression parameters for all 7289 proteins revealed that slope distributions were centered near one, while intercept distributions were near zero for all three cohorts. These were clearly distinguished from the mismatched cohort where the slope distribution centered around zero and the intercept centered around the measurement mean (Supplementary Figure S10A-B). This aligns with the correlation coefficient distributions which centered far above zero for cohorts A, B and C, indicating correlated signals, and near zero for the mismatched cohort, indicating no correlation (Supplementary Figure S10C).

**Fig. 5.**
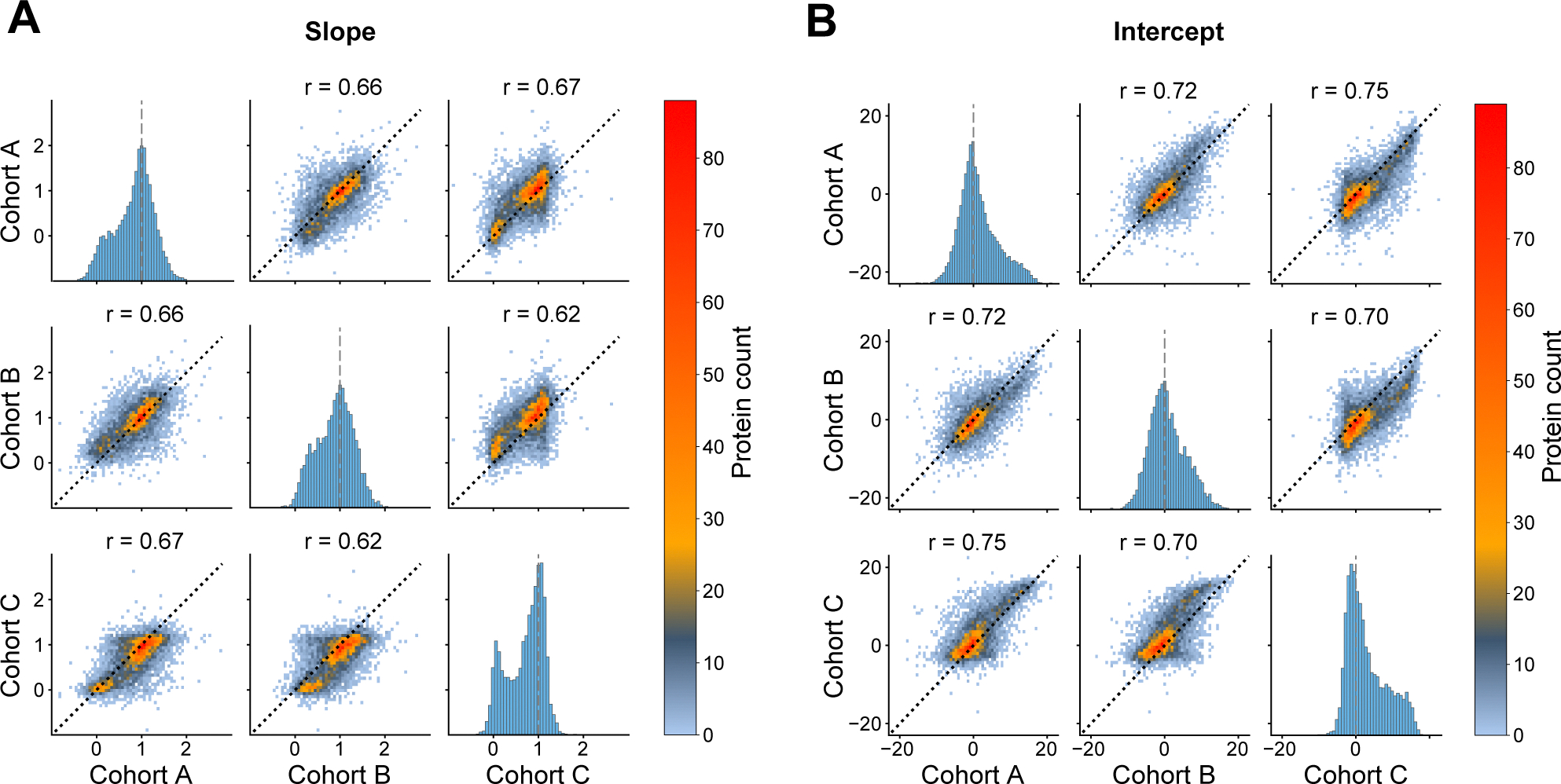
Inter-cohort agreement of scaling parameters. (A-B) Linear scaling parameters derived from cohorts A, B, and C were compared in a pairwise manner. Scaling parameters for all 7289 proteins were analyzed. Diagonal histograms represent the distribution of parameter values within each cohort. Off-diagonal 2D histograms depict between-cohort value distributions, with Pearson correlation coefficients (r) shown for each comparison. The black dotted line indicates perfect agreement between pairs. Linear regression slopes per protein are shown in A; the vertical dashed line denotes slope = 1 (indicating a similar scale for serum and plasma). Linear regression intercepts per protein are shown in B; the vertical dashed line denotes intercept = 0 (indicating the absence of a systematic bias between serum and plasma).

To explore the robustness of our serum-plasma scaling approach, we investigated the agreement between the linear regression-derived scaling parameters across the three cohorts. Pairwise comparisons revealed high consistency across all cohort pairs, as demonstrated by the high correlation coefficients for slopes (r = 0.62–0.67; Fig. 5A) and intercepts (r = 0.70–0.75; Fig. 5B). Furthermore, serum-plasma Spearman correlations at the protein level showed exceptionally high agreement between cohorts (r = 0.76–0.82; Supplementary Figure S11). The remarkable consistency across independent cohorts suggests that the scaling relationships between serum and plasma proteins are reproducible, supporting the generalizability of our scaling factors to new datasets.

### 3.4. Serum-to-plasma scaling of proteomic measurements preserves biomarker validity

**Fig. 6.**
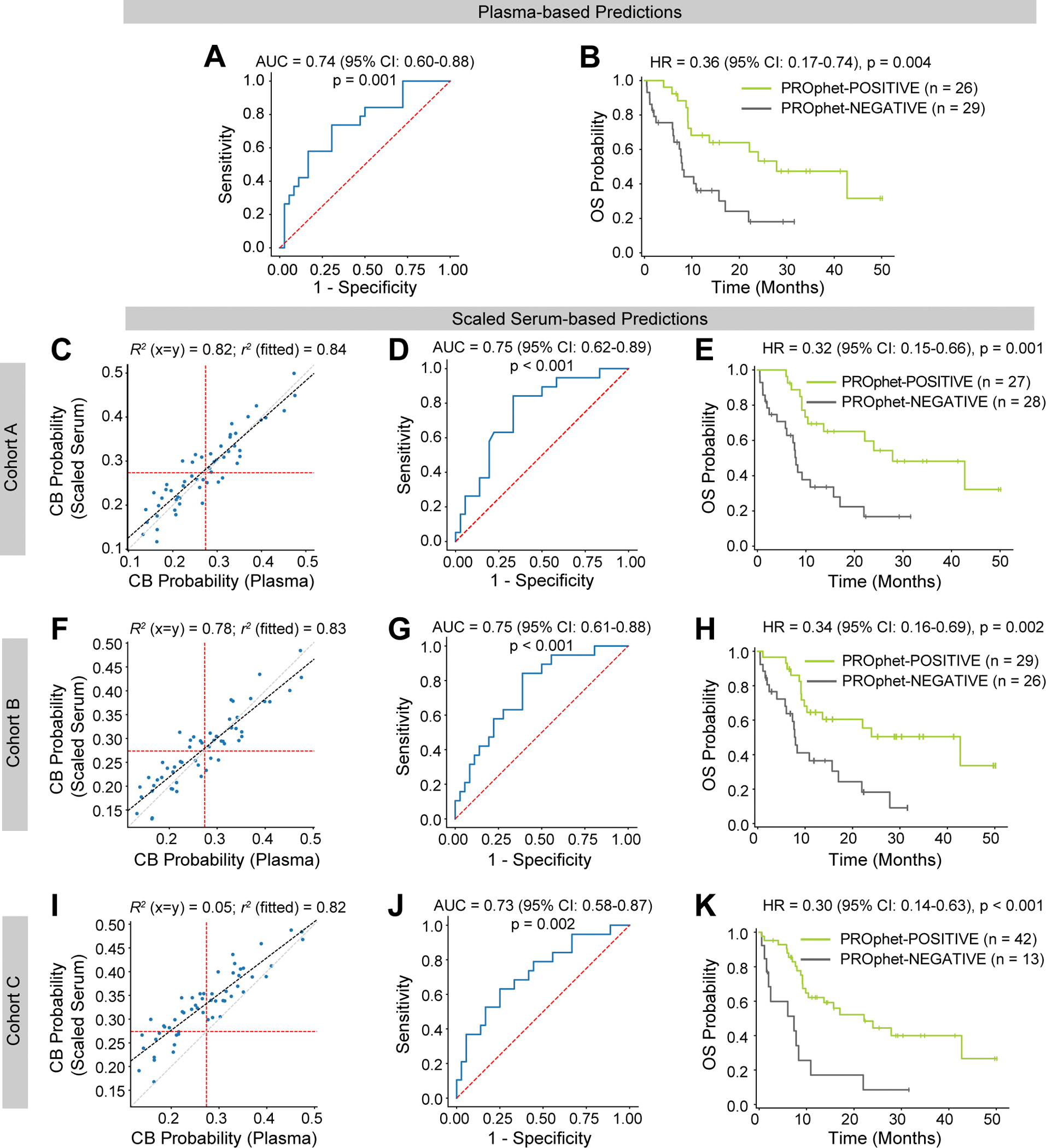
PROphet model performance based on plasma versus scaled-serum proteomic measurements. (A-B) Performance of the PROphet model was assessed using cohort A’s plasma protein dataset as input. Receiver Operating Characteristics (ROC) curve for clinical benefit (CB) prediction (A). Kaplan-Meier plot comparing overall survival (OS) of patients classified as PROphet-POSITIVE or PROphet-NEGATIVE (B). (C-K) The PROphet model was applied to cohort A serum proteomic data corrected with scaling factors derived from cohort A, cohort B or cohort C, as indicated. Plasma-based prediction of CB probability versus scaled serum-based prediction of CB probability. The gray dashed line indicates the identity line (x = y). The black dashed line indicates the best-fit line. Red dashed lines indicate the thresholds between PROphet-POSITIVE and PROphet-NEGATIVE groups (C, F, I). ROC curve for clinical benefit prediction (D, G, J). Kaplan-Meier plot comparing overall survival (OS) of patients classified as PROphet-POSITIVE or PROphet-NEGATIVE (E, H, K). AUC, area under the curve; HR, hazard ratio; CI, confidence interval; R^2^, agreement with the unity line; r^2^, agreement with the fitted line.

Since cohort A is composed of NSCLC patients treated with PD-1/PD-L1 inhibitors, it is the most relevant cohort (regarding cancer type and treatment type) to evaluate PROphet model performance. As expected, the model performed well on the cohort A plasma dataset, yielding an AUC of 0.74 and p-value = 0.001 (Fig. 6A). The model also successfully forecasted overall survival (OS) using this dataset. Specifically, OS was significantly longer in patients classified as PROphet-POSITIVE than those classified as PROphet-NEGATIVE (Fig. 6B; HR = 0.36, p = 0.004), consistent with previous reports [8].

Having confirmed that the model performs well with the cohort A plasma dataset, we tested model performance using cohort A serum proteomic data corrected with three different sets of scaling factors (i.e., derived from cohorts A, B, or C). Strong predictive performance was achieved when the serum data was corrected with scaling factors derived from cohorts A or B, as demonstrated by various measures. First, CB probabilities computed from the plasma dataset correlated strongly with CB probabilities calculated from either of the two scaled serum datasets (Figs. 6C and 6F; R^2^ = 0.82 for cohort A-derived scaling, R^2^ = 0.78 for cohort B-derived scaling). Second, there was substantial agreement between the plasma-based and scaled serum-based binary output of the model (Supplementary Table S13; 84 % and 87 % agreement, for cohort A and B, respectively, with p < 0.0001 in both cases). Third, AUCs from the scaled serum datasets were 0.75 with p < 0.001 in both cases (Figs. 6D and 6G), comparable to the plasma-based AUC (Fig. 6A). Fourth, the model stratified patients into two groups displaying significant differences in OS (Figs. 6E and 6H; cohort A-derived scaling: HR = 0.32, p = 0.001; cohort B-derived scaling: HR = 0.34, p = 0.002), similar to the plasma-based stratification (Fig. 6B). Interestingly, scaling factors derived from cohort C introduced a bias towards higher CB probabilities, resulting in weak agreement with the unity line (Fig. 6I; R^2^ = 0.05). This was expected, given that cohort C was flagged in our quality tests. Nevertheless, the scaled serum-based CB probabilities retained agreement with the fitted line, displaying r^2^ = 0.82. The predicted class agreement was lower than that achieved with cohort A- or B-derived scaling but still significant (Supplementary Table S13; 71 %, p < 0.0001). Despite this bias, the AUC measure and OS forecasting were comparable to those based on the plasma dataset (Figs. 6J and 6K), indicating that the bias in CB probability can be corrected via linear adjustment.

To quantify the effect of pre-analytical variability in bridging samples, we generated scaling factor sets from all possible cohort combinations and evaluated their effect on serum-to-plasma scaling validity in the context of PROphet performance. Median platelet contamination scores were calculated per bridging set. As expected, scores were most affected by the proportion of cohort C samples within the bridging set (Supplementary Figure S12A). In terms of PROphet performance, bridging sets with increasing contamination scores displayed decreasing agreement between plasma-based and scaled serum-based predictions, as measured by the R^2^ value. However, all other measures were unaffected (Supplementary Figure S12B-E) indicating that, in these cases, PROphet performance remains stable despite numerical biases introduced by pre-analytical variability.

To explore the generalizability of our scaling approach across different clinical characteristics, we employed three additional models developed on plasma proteomic data to predict patient sex, hypertension and COPD. Notably, there was minimal overlap between the biomarkers associated with each of the three models and the 388 PROphet biomarkers (Supplementary Figure S13). As before, cohort A serum proteomic data was corrected with the three different sets of scaling factors (i.e., derived from cohorts A, B, or C) and used as input for the three models. Strong predictive performance was achieved when the serum data was corrected with scaling factors derived from cohorts A, B or C, similar to plasma-based predictions (Supplementary Figure S14). These findings support the generalizability of our scaling approach.

In summary, predictions based on scaled serum data showed strong concordance with plasma-based predictions, even when the scaling factors were derived from unrelated cohorts (such as cohorts B and C representing diverse underlying diseases and sample collection protocols). These findings highlight the validity and robustness of our serum-plasma bridging method.

### 3.5. Key insights and practical guidelines

In biomarker research, the choice between serum or plasma as a sample matrix has long been debated, with concerns about their compatibility potentially hindering data integration from diverse cohorts. Our comprehensive analysis of 177 serum-plasma pairs across three cohorts provides new insights into this critical issue. Hierarchical clustering revealed a clear separation between serum and plasma samples. PCA showed that the first principal component, explaining 46 % of the total variability in the proteomic data, was attributable to specimen type. However, a deeper examination suggests that these substantial differences may be systematically reconcilable. We found that beyond the first principal component, subsequent components were not associated with specimen type but rather with cohort-specific factors, likely reflecting a combination of pre-analytical variables and biological differences. Remarkably, we found that for 91.6 % of the measured proteins, serum and plasma protein levels were correlated (p-value < 0.05). Notably, a comprehensive mass spectrometry-based analysis by Geyer et al. showed that only 299 of 2099 quantified proteins exhibit divergent expression levels across the two matrices, where proteins with the most significant differences were related to coagulation and platelet activation [24]. Taken together, we hypothesized that it may be possible to integrate serum and plasma datasets with careful statistical analysis and selective protein exclusion.

In this study, we developed a systematic approach for cross-specimen data utilization in the context of the plasma proteomics-based PROphet model. Various aspects underscore the suitability of this framework for serum-plasma bridging. First, the PROphet model is designed to be robust against measurement noise; it compensates for measurement variability by applying an additive approach, resulting in self-averaging of noise and signal amplification [8,20]. Second, the SomaScan assay offers measurement precision with a high dynamic range, representing a powerful biomarker development platform with broad applicability [29].

Using linear regression at the single protein level, we calculated scaling factors from serum-plasma pairs originating from three distinct cohorts. We found that each of the three scaling factor sets successfully adjusted serum proteomic measurements to their estimated plasma equivalents, as demonstrated by strong concordance between PROphet model predictions based on scaled serum data versus corresponding plasma-based predictions. While we observed some variations in numeric probability scores likely due to cohort-specific pre-analytical factors, the model’s ability to correctly rank patients by risk and predict clinical outcomes remained robust across the three sets of scaling factors. This robustness suggests that in cases where systematic differences are observed between cohorts, a simple calibration step could potentially be applied to align prediction scales while preserving the underlying biological signal.

Notably, scaling relationships between serum and plasma protein measurements were remarkably consistent across cohorts, suggesting that our scaling factors broadly apply to other datasets. Therefore, we provide scaling factors and median measurement values per protein from cohorts A, B and C for other researchers seeking to integrate serum and plasma datasets (Supplementary Tables S11 and S14). However, compatibility between our and future cohorts should be carefully considered to ensure valid cross-specimen bridging. Cohort compatibility can be assessed by comparing protein distributions between cohorts (e.g., using median deviation; Supplementary Table S11) per specimen type. The scaling factors from the most compatible cohort should be employed.

**Fig. 7.**
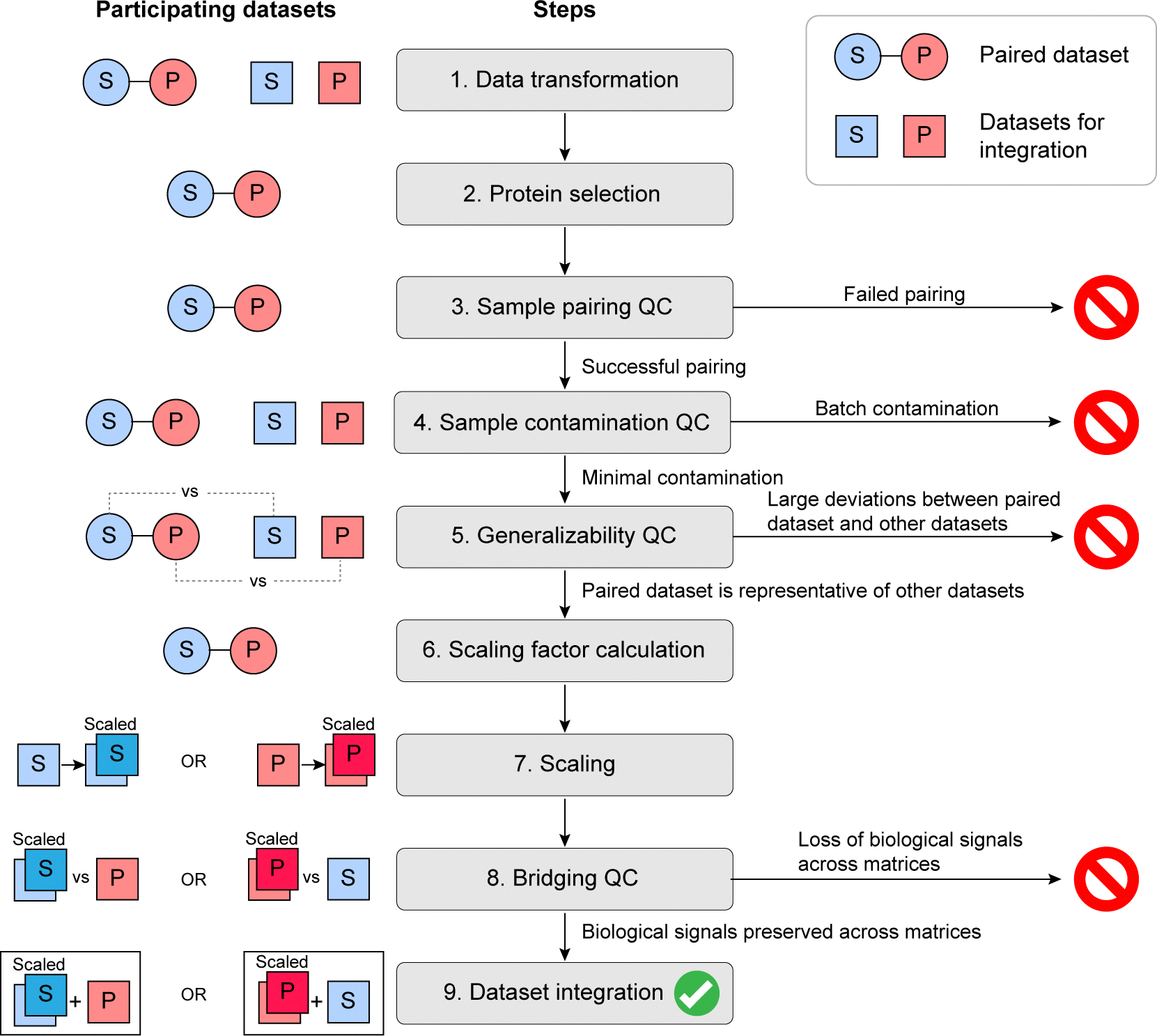
Serum-plasma scaling and integration. Flowchart of the serum-plasma dataset integration process, from raw measurements to the final integrated dataset. The process requires: (i) a paired serum-plasma dataset (from which scaling factors will be calculated); (ii) serum and plasma datasets for integration (to which scaling factors will be applied). Quality control (QC) steps serve as decision points for either continuing to the next step or pausing the process until the issue is addressed. P, plasma; S, serum.

For researchers interested in developing custom cross-specimen scaling factors, we propose a systematic process incorporating critical QC checkpoints to ensure robust and reliable results (Fig. 7). The process requires a paired serum-plasma dataset from which to calculate scaling factors and datasets for integration to which the scaling factors will be applied. The process is as follows:

- Data transformation: Given the skewed (roughly log-normal) distributions characteristic of proteomic data, log-transformation should be applied to the raw measurements across all datasets to mitigate the effects of outliers and facilitate the application of parametric methods (e.g., linear regression).
- Protein selection: Correlations between serum and plasma measurements per protein should be assessed in the paired serum-plasma dataset. Proteins with low serum-plasma correlations should be excluded from joint specimen analysis. This is a crucial step for ensuring reliable cross-specimen bridging later on. As a minimum requirement, proteins with non-significant Spearman correlation (q < 0.05 after Benjamini–Hochberg adjustment) should be excluded. However, higher thresholds may be necessary depending on model development needs. For example, applying the Spearman correlation filter to Cohort A’s serum-plasma dataset would exclude 1497 proteins after Benjamini-Hochberg adjustment (Supplementary Table S14). However, the threshold Spearman correlation for significance is low (Spearman r ~ 0.28). Thus, a higher threshold is advisable in this case.
- Sample pairing QC: The paired serum-plasma dataset should beevaluated regarding agreement between sample pairs. Sample pairs displaying low correlation should be excluded from the pipeline before proceeding (as described in Materials and Methods, Equation 1). If paired samples are generally not well correlated, it is advisable to halt the process, as serum-plasma bridging will not be valid.
- Sample contamination QC: To avoid introducing bias, sample contamination in all samples should be identified and addressed. Platelet and erythrocyte contamination can be evaluated using previously described proteomic marker panels [24]. If available, high-quality external samples may be positive controls per specimen type. Contaminated samples can be removed from the pipeline before proceeding. However, if an entire batch is contaminated, it may be advisable to halt the process or acquire new samples.
- Generalizability QC: Scaling factors calculated from the paired dataset will apply to the measurements of interest if the paired dataset is representative of these measurements. To confirm generalizability, agreements between the measurement distribution per protein in the paired dataset versus those in the measurements of interest should be assessed. For example, plasma protein medians from the paired dataset should correlate with plasma protein medians from the measurements of interest; the same should apply to serum protein medians. Large deviations imply that the scaling factors will not apply to the measurements of interest. Notably, weak agreement between the compared sets may be due to differences in sample handling. Thus, technical issues should be considered when interpreting results.
- Scaling factor calculation: If all QC measures passed until this point, protein-specific scaling factors can be calculated from the paired dataset using an appropriate function (e.g., linear regression or others). Simple functions require fewer paired samples as they are less likely to overfit.
- Scaling: Scaling factors can now be applied to the relevant datasets to scale measurements from one specimen type (e.g., serum) to equivalents of the other specimen type (e.g., plasma).
- Bridging QC: To validate bridging success, we recommend a final QC step that evaluates whether the biological signals of interest are preserved across specimen types after scaling. The appropriate validation approach depends on the downstream biological analysis. Examples include: (a) For predictive modeling, confirm that classifiers trained on one specimen type maintain performance when applied to scaled measurements of the other specimen type. (b) For descriptive or exploratory analyses, verify that individual protein measurements or expression profiles show adequate agreement between specimen types, e.g., by verifying that sample pairs cluster together. (c) For pathway or enrichment analyses, confirm that biological process annotations and pathway memberships remain stable between specimen types, such that proteins enriched in a biological process in one matrix are similarly enriched after scaling. Finally, the protein selection thresholds may need to be adjusted (step ii) based on the required level of agreement and the stability of the biological measure.

### 3.6. Strengths and limitations

To the best of our knowledge, only one other report describes serum-plasma bridging methodology. The study developed a tiered linear modelling approach using 1463 Olink-quantified proteins across matched serum and plasma samples [16]. In comparison, our study surveys a substantially larger set of proteins (7289 versus 1463 proteins) using the SomaScan platform. Our approach minimizes bias through various QC measures addressing contamination and compatibility issues. Importantly, we validate our scaling approach in the context of a clinically-relevant predictive model (PROphet). Together, these studies demonstrate that serum-plasma bridging is achievable across different platforms. Our study has some limitations that should be acknowledged. First, the analysis was limited to SomaScan proteomic data. Second, non-cancer diseases and healthy controls were not explored which may limit generalizability. Despite these limitations, our study provides a valuable framework for integrating serum and plasma proteomic datasets, facilitating cross-comparison of protein measurements from diverse clinical studies and biobanks that specimen type differences have historically limited.

## 4. Conclusions

In this study, we describe a systematic approach for bridging between serum and plasma proteomic measurements. The approach is based on linear scaling factors computed from matched serum-plasma proteomic dataset pairs that have passed compatibility and quality checks. We validate the bridging process in the context of a plasma proteomics-based predictive model and provide practical guidelines for integrating serum and plasma datasets. Our study contributes to the arsenal of tools enabling cross-specimen data utilization. Such approaches could help unlock previously incompatible datasets for joint analysis, ultimately accelerating biomarker discovery and validation efforts.

## Supplementary Material

Supplementary Material - JPBA

Supplementary Table S9

Supplementary Table S10

Supplementary Table S12

Supplementary Table S13

Supplementary Table S8

Supplementary Table S11

Supplementary Table S14

## Data Availability

The data supporting the findings of this study are available in the supplemental material. The individual-level data used in this research was collected in a real-world healthcare setting and as such is subject to controlled access due to privacy considerations. Public availability is restricted by the ethics committee and/or informed consent requirements. OncoHost may enable access to some additional data following a signed data use agreement. For data access requests, please contact the corresponding author.

## Code availability

Python code for generating manuscript figures, for performing iterative scaling and outlier detection, and a comprehensive list of Python packages used are available at: https://github.com/OncoHostPlatform/SERUM-PLASMA-BRIDGING-2025

## Appendix A. Supporting information

Supplementary data associated with this article can be found in the online version at doi:10.1016/j.jpba.2026.117421.
