# Supplementary material for: Bridging the gap: A systematic approach to integrating serum and plasma proteomic datasets for biomarker studies
Source: J Pharm Biomed Anal. Author manuscript; Available in PMC 2026 Mar 23. (PMC13008497; doi:10.1016/j.jpba.2026.117421)
Supplement: Supplementary Material - JPBA [file NIHMS2148897-supplement-Supplementary_Material_-_JPBA.docx]

**Supplementary Data**

**Bridging the gap: A systematic approach to integrating serum and plasma proteomic datasets for biomarker studies**

Coren Lahav^a,*^, Nili Dahan^a^, Michal Harel^a^, Yehonatan Elon^a^, Itamar Sela^a^, Philipp E. Geyer^b^, Marc A. Schneider^c,d^, Thomas Muley^c,d^, Antonella Bacchiocchi^e^, Jennifer L. Marte^f^, Charalampos S. Floudas^f^, Ruth Halaban^e^, Mario Sznol^g^, Petros Christopoulos^c,h^, James L. Gulley^f^

Supplementary Tables

**Supplementary Table S1A: Summary of cohorts**

| **Cohort** | **Disease (n)** | **Patient count** | **Sample count** | **Sample matrix** | **Time points** |
| --- | --- | --- | --- | --- | --- |
| **A** | NSCLC | 55 | 55 x 2 | Paired serum and plasma | Pre-treatment |
| **B** | HPV-related malignancies | 44 | 82 x 2 | Paired serum and plasma | Pre-treatment (n=43)  On-treatment (n=39) |
| **C** | Melanoma | 47 | 47 x 2 | Paired serum and plasma | Pre-treatment |
| **Plasma reference** | NSCLC (n=445),  Melanoma (n=88) | 533 | 533 | Plasma | Pre-treatment |
| **Serum reference** | Melanoma (n=59),  HPV-related malignancies (n=44) | 103 | 103 | Serum | Pre-treatment |

HPV, human papillomavirus; NSCLC, non-small cell lung cancer

**Supplementary Table S1B: Institutional Review Board (IRB) reference numbers per medical center providing plasma or serum samples**

| **Medical center** | **IRB reference number** |
| --- | --- |
| Thorax Clinic, Heidelberg University Hospital | S-270/2001 |
| National Cancer Institute | 18-C-0056, 20-C-0045 |
| Smilow Cancer Hospital-Yale New Haven Health | IRB Protocol ID: 609001869 |
| Roswell Park Comprehensive Cancer Center  (Bio Bank) | MODCR00001188 / I-03103 |
| Roswell Park Comprehensive Cancer Center (PROPHETIC site) | CR00006374 / P-1646021 |
| Asklepios Klinik Gauting GmbH, Asklepios Biobank for Lung Diseases | 21-0279 |
| Sheba Medical Center | 6349-19-SMC |
| Hadassah Medical Center | HMO-0389-19, MOH - 202014059 |
| Cheltenham General Hospital | IRAS project ID: 290564,  REC reference 21/EM/0055 |
| Sylvester Comprehensive Cancer Center, University of Miami | 20201014 |
| Rambam Medical Center | RMB-0456-19 |
| Rabin Medical Center | 0038-20-RMC, MOH - 202015744 |
| Haemek Medical Center | 0178-19-EMC, MOH -202014653 |
| Shamir Medical Center | 0294-20-ASF, MOH - 202119884 |
| Kaplan Medical Center | 0183-19-KMC, MOH - 202015964 |
| Soroka Medical Center | 0424-19-SOR, MOH -202016280 |
| Rutgers Cancer Institute | 20203501 |
| Bnai Zion Medical Center | 0091-19-BNZ, MOH - 201913401 |
| Aberdeen Royal Infirmary | IRAS project ID: 290564,  REC reference 21/EM/0055 |
| Meir Medical Center | 0240-19-MMC |
| Barzilai Medical Center | 0100-19-BRZ, MOH - 202120373 |
| Royal Bournemouth General Hospital Dorset | IRAS project ID: 290564,  REC reference 21/EM/0055 |
| Singleton Hospital Cancer Institute, Swansea | IRAS project ID: 290564,  REC reference 21/EM/0055 |
| Tel Aviv Sourasky Medical Center | 0495-19-TLV, MOH - 201913104 |
| Mount Vernon Cancer Centre | IRAS project ID: 290564,  REC reference 21/EM/0055 |
| Assuta Medical Centers | 0011-20-ASMC |
| Mayo Clinic Jacksonville | 21-008192 |
| Florida Cancer Specialists & Research Institute | 20203501 |

**Supplementary Table S2: Patient characteristics – Cohort A (NSCLC)**

| **N** |  | 55 |
| --- | --- | --- |
| **Age, mean (SD)** |  | 63.6 (8.7) |
| **Sex, N (%)** | Female  Male | 18 (32.7)  37 (67.3) |
| **Histology, N (%)** | Adenocarcinoma  Squamous cell carcinoma  Other | 37 (67.3)  13 (23.6)  5 (9.1) |
| **PD-L1, N (%)** | ≥50%  1-49%  <1% | 21 (38.2)  19 (34.5)  14 (25.5) |
| **Treatment type, N (%)** | Anti-PD1 +chemotherapy  Anti-PD1  Anti-PDL1 | 37 (67.3)  17 (30.9)  1 (1.8) |
| **Treatment line, N (%)** | First | 55 (100) |

NSCLC, non-small cell lung cancer

**Supplementary Table S3: Patient characteristics – Cohort B (HPV-related malignancies)**

| **N** |  | 44 |
| --- | --- | --- |
| **Age, mean (SD)** |  | 54.4 (13.4) |
| **Sex, N (%)** | Female  Male | 32 (72.7)  12 (27.3) |
| **Indication, N (%)** | Anogenital  Cervical  Head and neck squamous cell carcinoma | 17 (38.6)  18 (40.9)  9 (20.5) |
| **Treatment type, N (%)** | Bintrafusp Alfa (a bifunctional fusion protein targeting TGF-β and PD-L1) | 44 (100) |
| **Treatment line, N (%)** | Unknown | 44 (100) |

HPV, human papillomavirus

**Supplementary Table S4: Patient characteristics – Cohort C (Melanoma)**

| **N** |  | 47 |
| --- | --- | --- |
| **Age, mean (SD)** |  | 60.5 (12.6) |
| **Sex, N (%)** | Female  Male | 13 (27.7)  34 (72.3) |
| **Histology, N (%)** | Cutaneous  Acral  Mucosal  Unknown | 32 (68.1)  7 (14.9)  6 (12.8)  2 (4.3) |
| **Treatment type, N (%)** | Anti-PD1 + anti-CTLA4  Anti-PD1  Anti-CTLA4 | 27 (57.4)  17 (36.2)  3 (6.4) |
| **Treatment line, N (%)** | First  Advanced  Unknown | 27 (57.4)  19 (40.4)  1 (2.1) |

**Supplementary Table S5: Comparison of plasma sample preparation protocols**

|  | **Cohort A** | **Cohort B** | **Cohort C** |
| --- | --- | --- | --- |
| **Tube type** | EDTA | EDTA | EDTA |
| **Tube vendor,**  **catalogue number** | S-Monovette® (SARSTEDT) EDTA K3E  Cat # 01.1605.001 | BD Vacutainer K2E Cat # 366643 | BD Vacutainer K2E  Cat # 367841 |
| **Time to spin** | 5 – 60 min  (median 30 min) | Within 36 h; kept at room temp until processing | Within 15-30 minutes |
| **Spin #1 time** | 10 min | 10 min | 10 min |
| **Spin #1 temperature** | 20° C | Room temperature | 18°C |
| **Spin #1 speed** | 2,000 g | 495 g | 800 g |
| **Spin #2 time** | N/A | 10 min | 10 min |
| **Spin #2 temperature** | N/A | Room temperature | Room temperature |
| **Spin #2 speed** | N/A | 1,170 g | 450 g |
| **Storage temperature** | -80°C | -80°C | -80°C |

**Supplementary Table S6: Comparison of serum sample preparation protocols**

|  | **Cohort A** | **Cohort B** | **Cohort C** |
| --- | --- | --- | --- |
| **Tube type** | Serum separator tube | Serum separator tube | Serum separator tube |
| **Tube vendor,**  **catalogue number** | S-Monovette® (SARSTEDT) Serum Gel CAT  Cat # 01.1602 | Greiner Bio-One Vacutainer  Cat #455071 | BD Vacutainers  Cat # 367820 |
| **Time to spin** | Up to 1 h | Within 36 h. If the time between sample draw and receipt was >4 hrs, the specimen was refrigerated. | Within 15-30 minutes |
| **Spin time** | 10 min | 10 min | 10 min |
| **Spin temperature** | 20°C | 4°C | 18°C |
| **Spin speed** | 2,000 g | 1,170 g | 800 g |
| **Storage temperature** | -80°C | -80°C | -80°C |

**Supplementary Table S7: Trait frequency distribution in development and validation sets**

|  | |  | | **PROphet model**  **development set (N=228)** | | **Cohort A:**  **Validation set (N=55)** |
| --- | --- | --- | --- | --- | --- | --- |
| **Sex, n (%)** | Female | | 76 (33.3%) | | 18 (32.7%) | |
|  | Male | | 152 (66.7%) | | 37 (67.3%) | |
| **Hypertension, n (%)** | Positive | | 45 (19.7%) | | 12 (21.8%) | |
|  | Negative | | 183 (80.3%) | | 43 (78.2%) | |
| **COPD, n (%)** | Positive | | 31 (13.6%) | | 5 (9.1%) | |
|  | Negative | | 197 (86.4%) | | 50 (90.9%) | |

COPD, chronic obstructive pulmonary disease

Supplementary Tables S8-S14 are provided as excel files.

Titles are listed below:

| **Table number and title** | File name |
| --- | --- |
| **Supplementary Table S8: Serum-plasma correlation** | mmc2.xlsx |
| **Supplementary Table S9: Enrichment analysis of proteins with extreme plasma-serum ratios** | mmc3.xlsx |
| **Supplementary Table S10: Enrichment analysis of proteins categorized by serum-plasma correlation strength** | mmc4.xlsx |
| **Supplementary Table S11: Medians and difference between internal and external cohorts** | mmc5.xlsx |
| **Supplementary Table S12: Enrichment analysis of concordance outliers** | mmc6.xlsx |
| **Supplementary Table S13: Contingency table comparing PROphet binary classification based on plasma versus scaled serum protein measurements** | mmc7.xlsx |
| **Supplementary Table S14: Scaling factors by cohort** | mmc8.xlsx |

Supplementary Figures


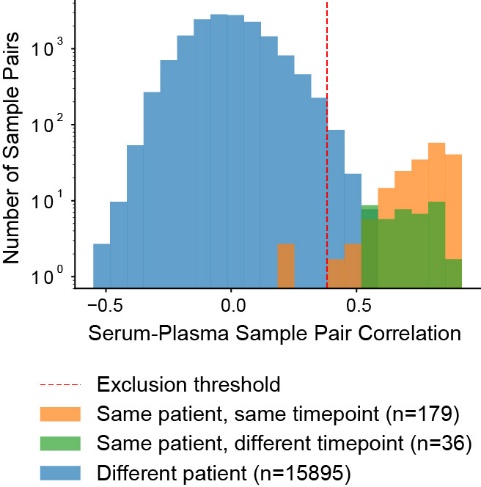


**Supplementary Figure S1: Sample pairing.** The analysis was performed on 388 proteins, corresponding to the set of PROphet proteomic biomarkers. Pearson correlation between serum-plasma pairs (orange), samples collected from the same patient at different timepoints (green), and all possible unpaired, independent sample combinations (blue). The red dashed line indicates the sample pairing exclusion criterion. Two sample pairs (same patient, same timepoint) fell below this threshold and were therefore excluded.


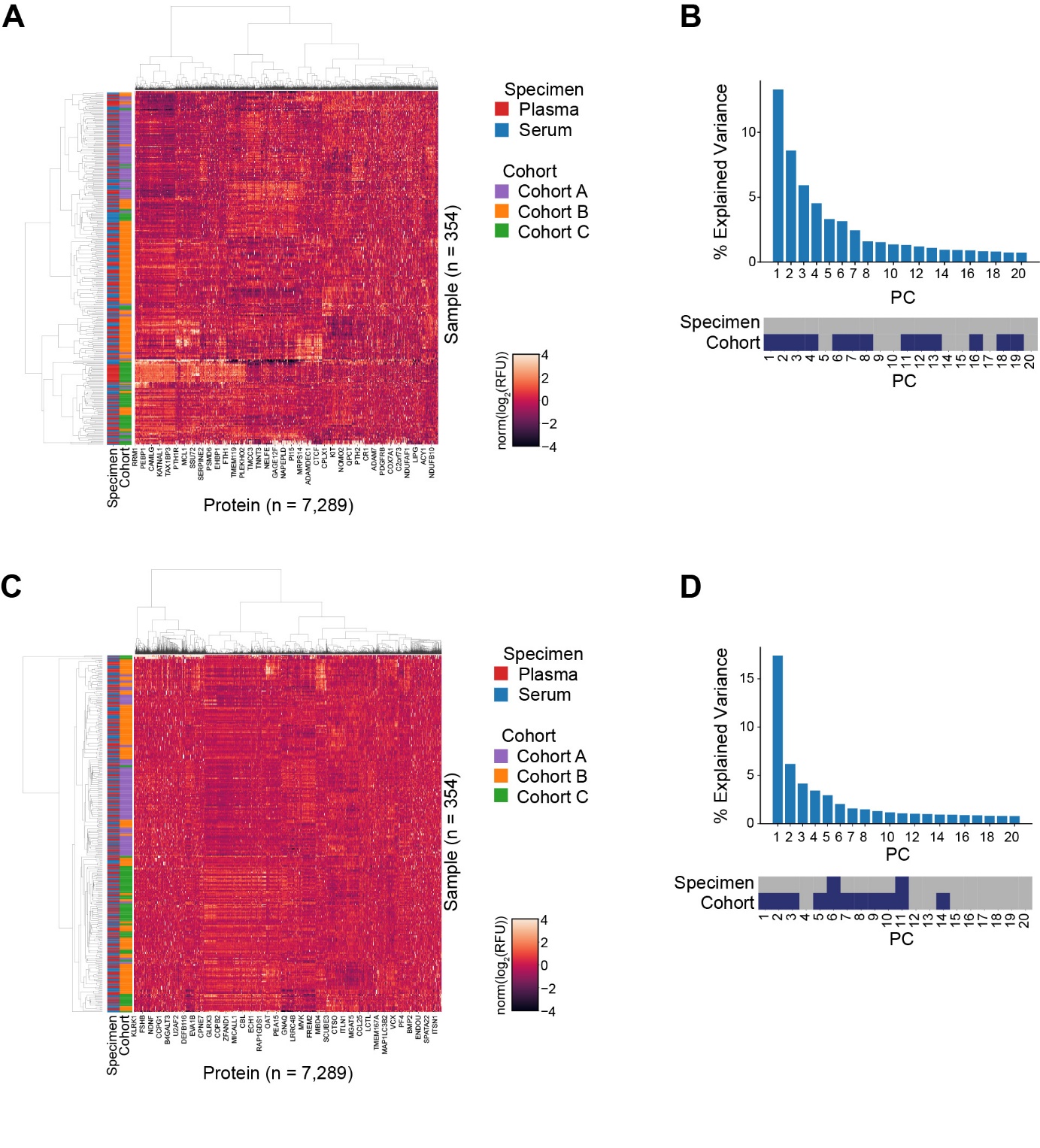


**Supplementary Figure S2: Iterative and robust scaling.** Serum and plasma protein measurements were normalized separately using an iterative (A-B) or robust (C-D) scaling method. (A,C) Hierarchical clustering of normalized log_2_-transformed protein measurements. (B,D) Principal component analysis. Top panel: The bar graph shows the percentage of explained variance by the top 20 principal components (PCs). Bottom panel: Association of the top 20 PCs with specimen type and cohort (bottom). Blue, p < 0.05; gray, not significant. The specimen-associated component (first PC in Figure 2) is lost when serum and plasma protein measurements are normalized separately.


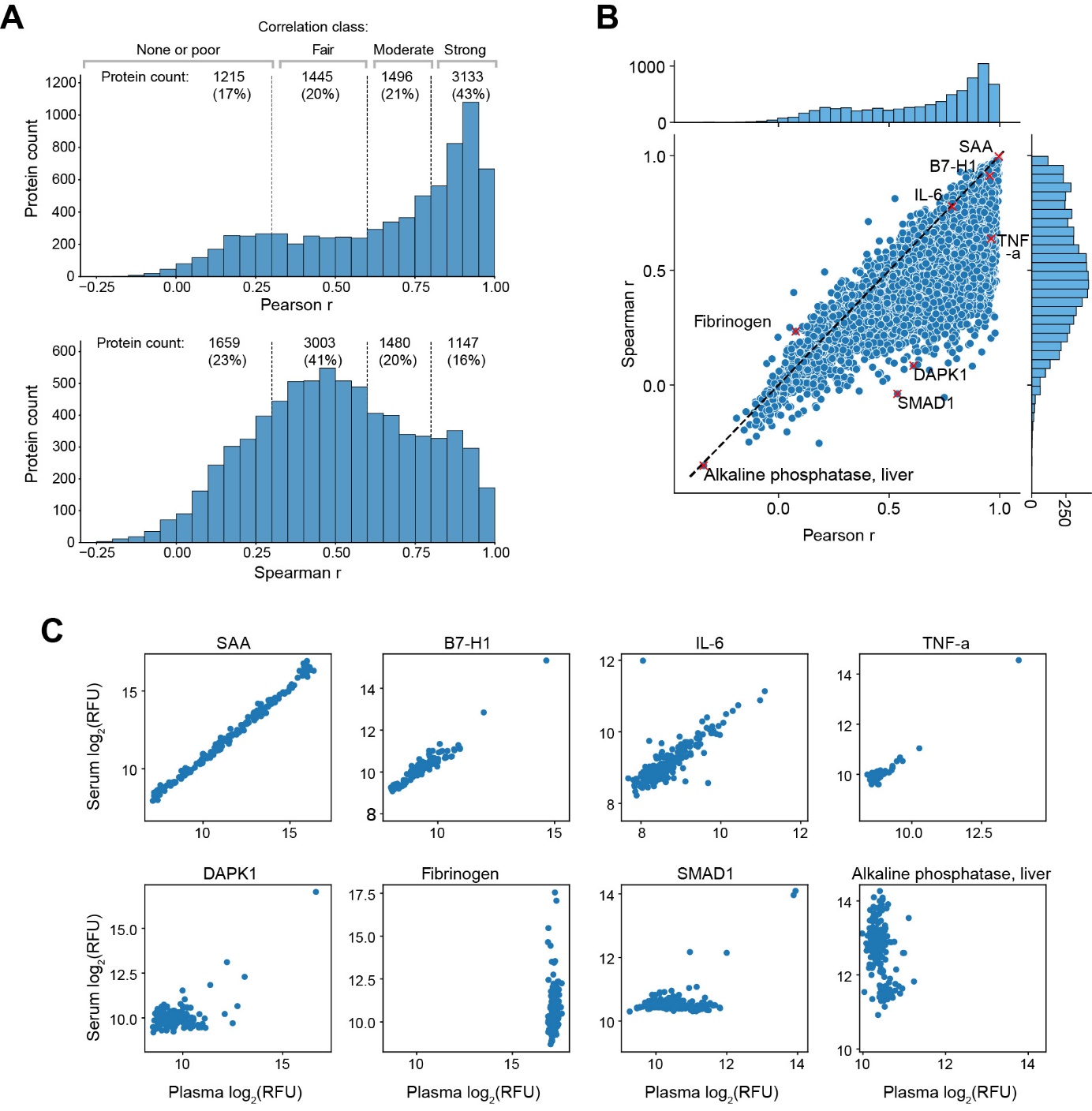


**Supplementary Figure S3: Correlations between plasma and serum proteomes.** (A) Plasma-serum protein expression correlation histograms (top: Pearson correlation, bottom: Spearman correlation). Correlation classes are based on [1]. Protein count per correlation class is shown. (B) Plasma-serum protein expression correlation scatter plot and histograms (x, top: Pearson correlation, y, right: Spearman correlation). Each dot represents a protein. The black dashed line indicates equality between correlation methods. (C) Plasma-serum measurement value scatterplots for proteins labeled in B. Analyses presented throughout the figure are based on the entire set of 7,289 protein analytes across three cohorts (cohorts A, B, and C).

[1] Y.H. Chan, Biostatistics 104: correlational analysis, Singapore Med J 44(12) (2003) 614-9.


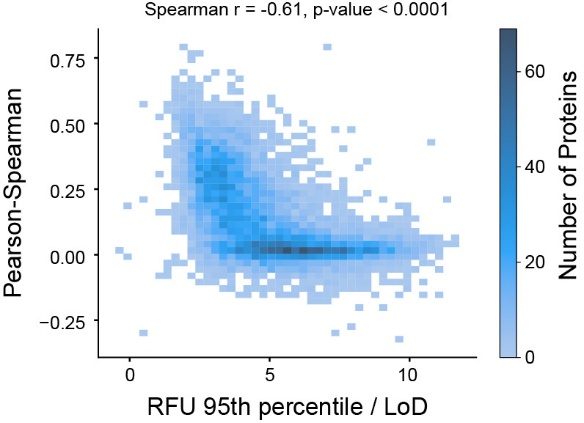


**Supplementary Figure S4: Relationship between Pearson-Spearman correlation differences and protein measurability.** Bivariate histogram showing the negative correlation (Spearman r = -0.61, p < 0.0001) between protein measurability, represented by the RFU 95th percentile / LoD ratio (x-axis) and the difference in Pearson and Spearman correlations (y-axis). RFU, relative fluorescence units; LoD, limit of detection, defined as the median signal of the negative control samples plus 3*standard median absolute deviation of the negative control. All 7,289 protein analytes were included in the analysis.


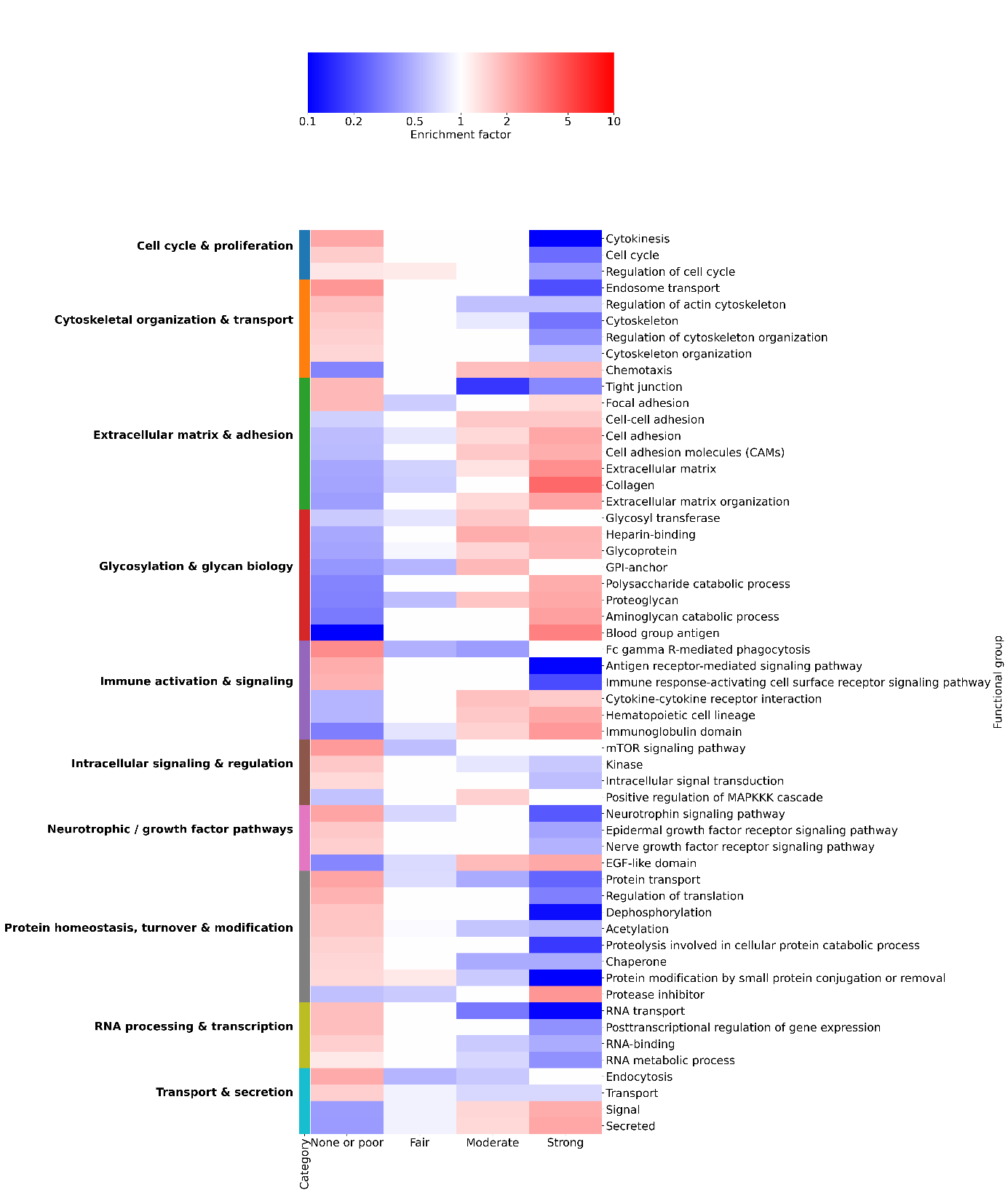


**Supplementary Figure S5:** **Functional enrichment analysis of proteins grouped by serum-plasma correlation strength.** Heatmap showing enrichment factors of selected functional categories for the different serum-plasma correlation sets: strong (r ≥ 0.8), moderate (0.6 ≤ r < 0.8), fair (0.3 ≤ r < 0.6), or none/poor (r < 0.3). Each row represents a specific biological function or pathway, organized into broader functional categories (left margin). The color scale indicates the enrichment factor, with blue representing depletion (< 1-fold), white representing no enrichment (1-fold), and red representing enrichment (> 1-fold) relative to the background distribution. Enrichment analysis was performed using Fisher's exact test; only categories with significant enrichment (Benjamini-Hochberg adjusted q < 0.1) are shown with their calculated enrichment factor, while non-significant categories (q > 0.1) are displayed as 1-fold (white).


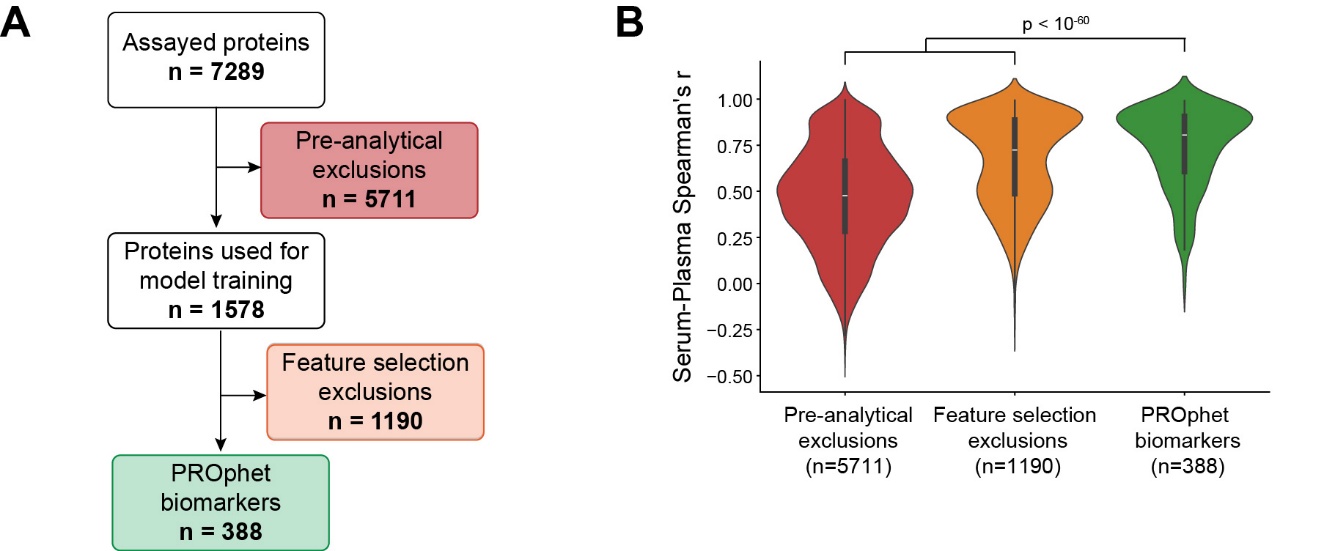


**Supplementary Figure S6: Serum-plasma Spearman correlation distributions of protein measurements in different protein sets.** (A) PROphet model development involved two protein filtering steps: (i) pre-analytical filtering reduced the set of 7,289 proteins to a subset of 1,578 proteins with high stability across different plasma separation methods; (ii) feature selection resulted in a set of 388 proteins serving as predictive biomarkers. (B) Violin plots displaying serum-plasma Spearman correlation distributions for the two excluded protein sets (n=5,711; n=1,190) and the biomarker set (n=388). The analysis was performed on combined cohort A, B, and C datasets.


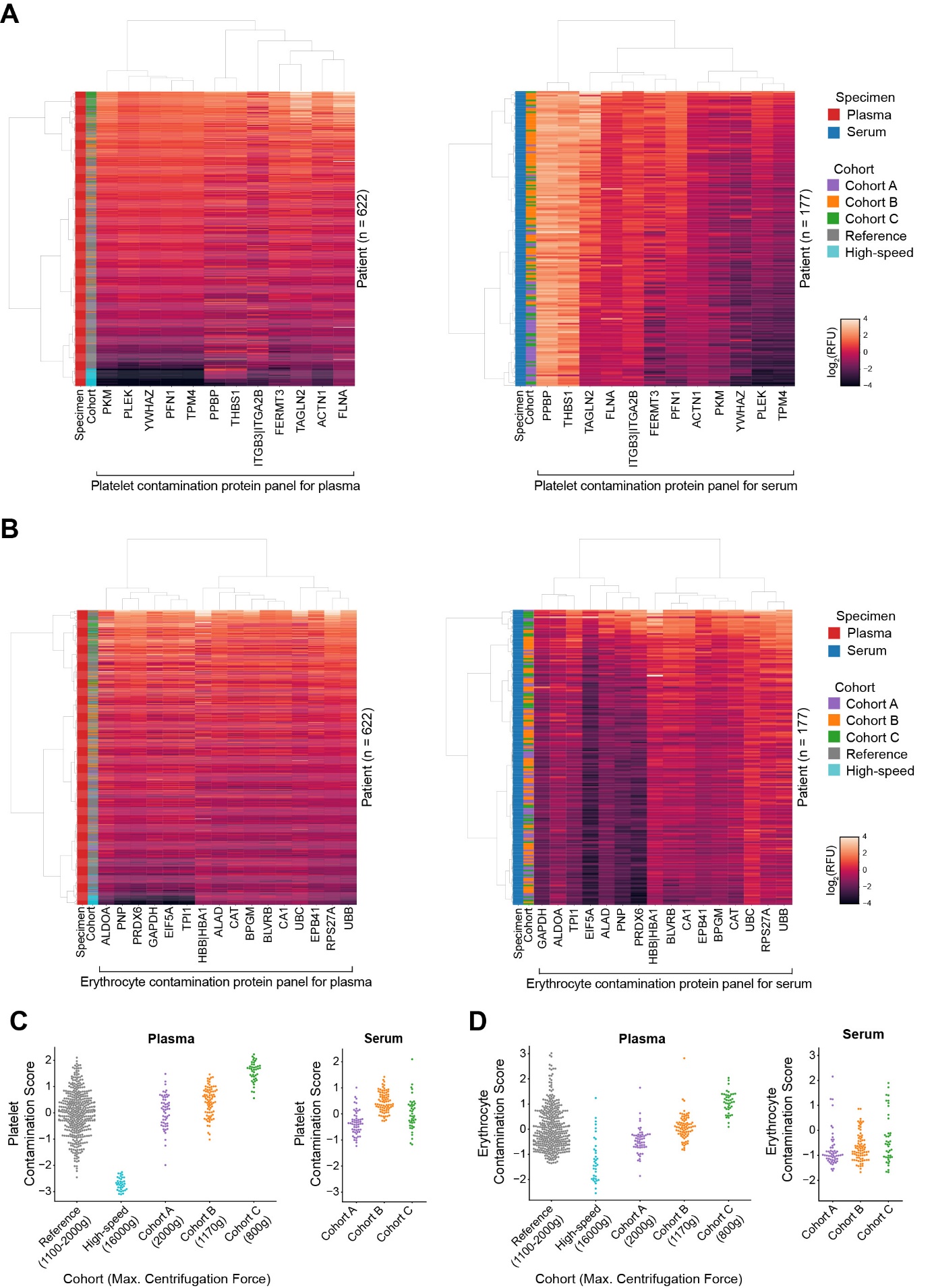


**Supplementary Figure S7: Assessment of platelet and erythrocyte contamination.** Plasma and serum proteomic datasets from cohorts A, B, and C were normalized via iterative z-scaling against a reference plasma dataset from an external cohort of 409 patients with NSCLC. (A-B) Heatmap representations of normalized expression levels of platelet contamination markers (A) and erythrocyte contamination markers (B) in plasma and serum samples from cohorts A ,B, and C. The heatmaps for plasma samples also include (i) reference plasma samples (n=409) and (ii) an external set of plasma samples prepared with high-speed centrifugation (16,000g) serving as a positive control for minimal platelet contamination (n=36). (C-D) Platelet contamination scores (C) and erythrocyte contamination scores (D) for plasma and serum samples were calculated by averaging normalized measurements of the relevant markers per patient.


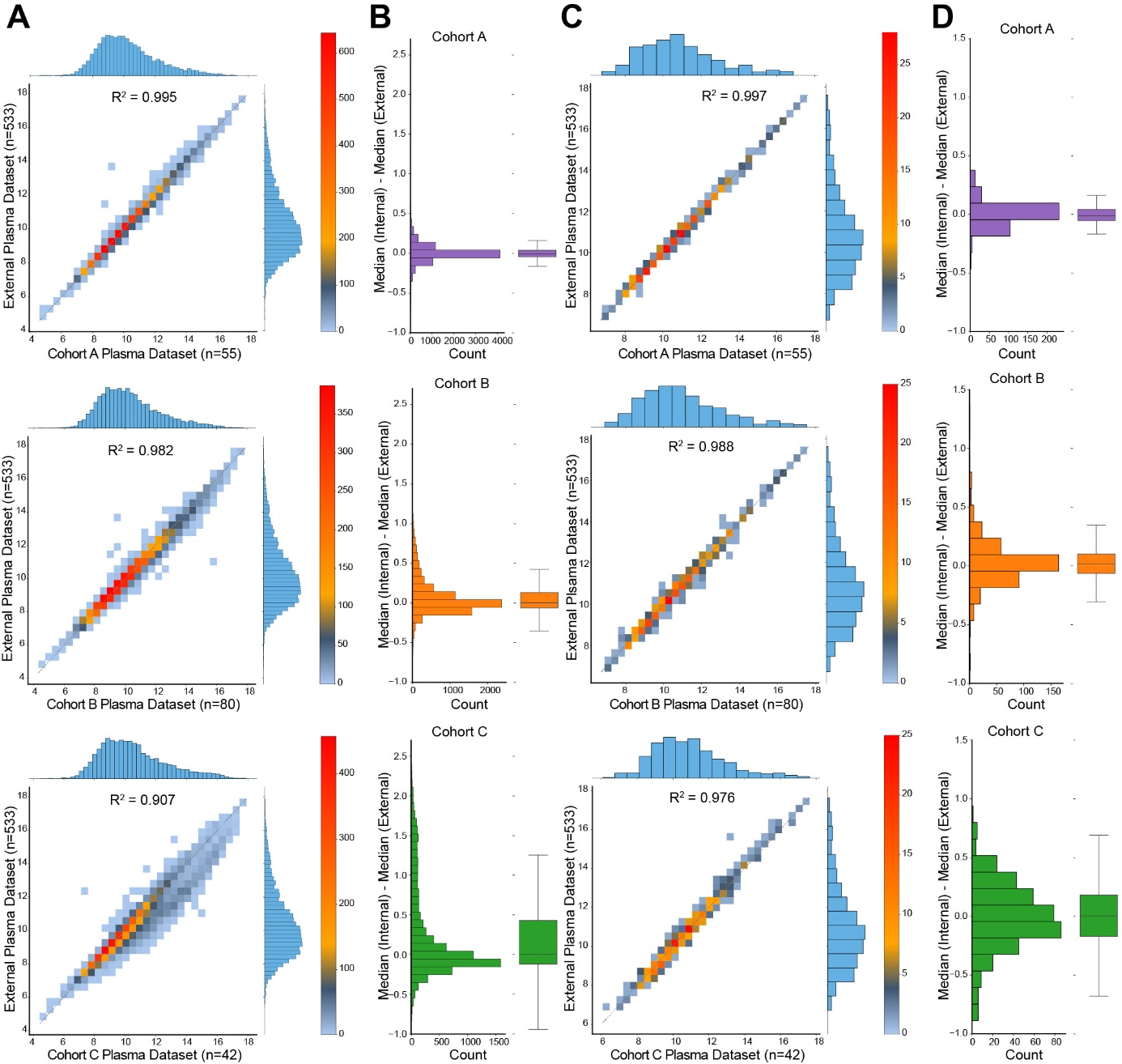


**Supplementary Figure S8: Concordance between internal and external plasma datasets.** Plasma datasets from cohorts A, B, and C were compared against an external plasma dataset (based on 445 NSCLC patients and 88 melanoma patients). (A) Correlation between median values (per protein) in internal and external datasets was evaluated. The coefficient of determination (R^2^) is shown for each comparison. The analysis was performed for all 7,289 proteins. (B) Histograms and boxplots showing distances from the diagonals in A, representing differences in medians between internal and external plasma datasets. (C-D) As in A and B, performed on 388 PROphet proteomic biomarkers.


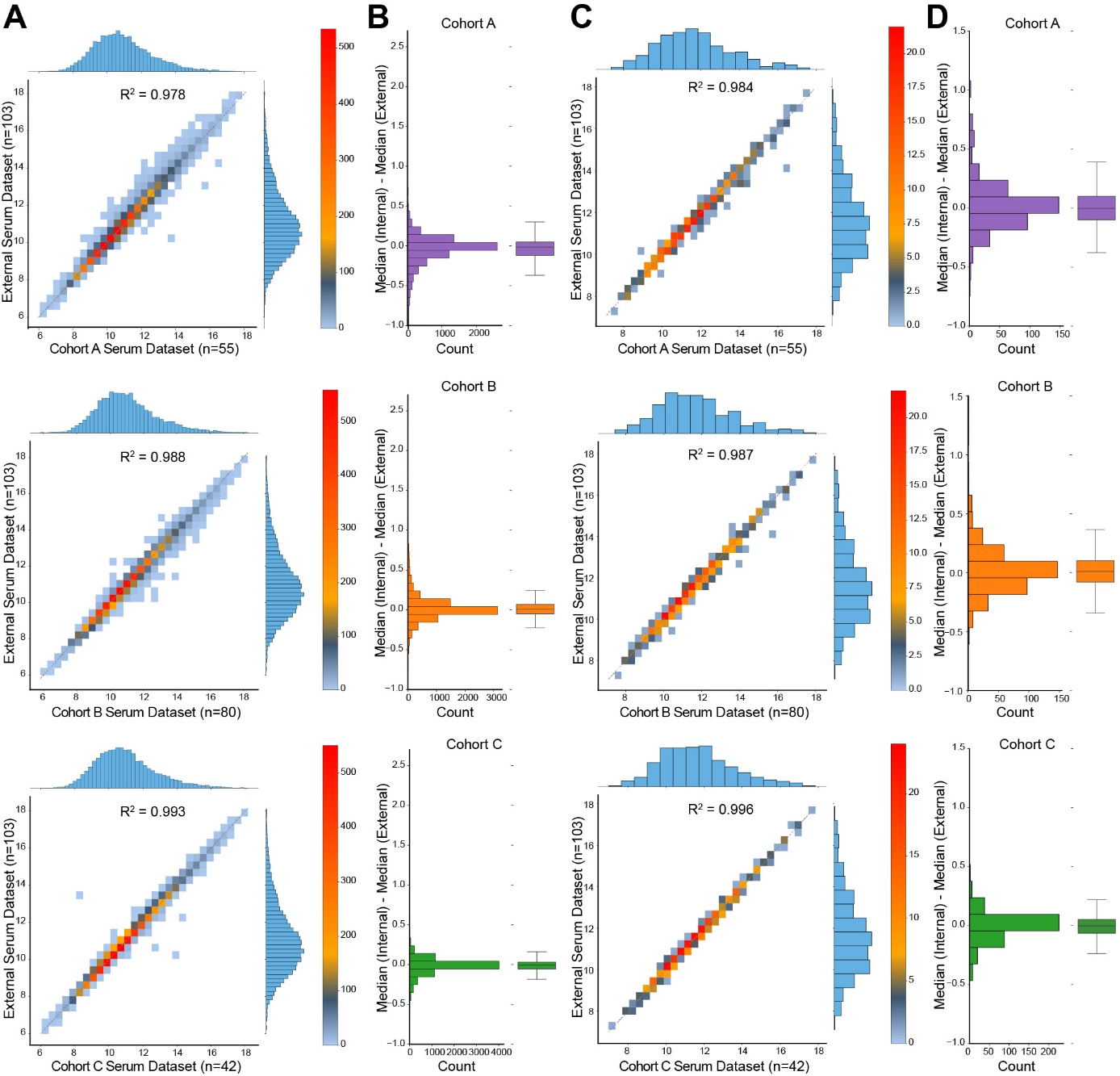


**Supplementary Figure S9:** **Concordance between internal and external serum datasets.** Serum datasets from cohorts A, B, and C were compared against an external serum dataset (based on 59 melanoma patients and 44 patients with HPV-related malignancies). (A) Correlation between median values (per protein) in internal and external datasets was evaluated. The coefficient of determination (R^2^) is shown for each comparison. The analysis was performed for 7,289 proteins. (B) Histograms and boxplots showing distances from the diagonals in A, representing differences in medians between internal and external serum datasets. (C-D) Same as in A and B, performed on 388 PROphet proteomic biomarkers.


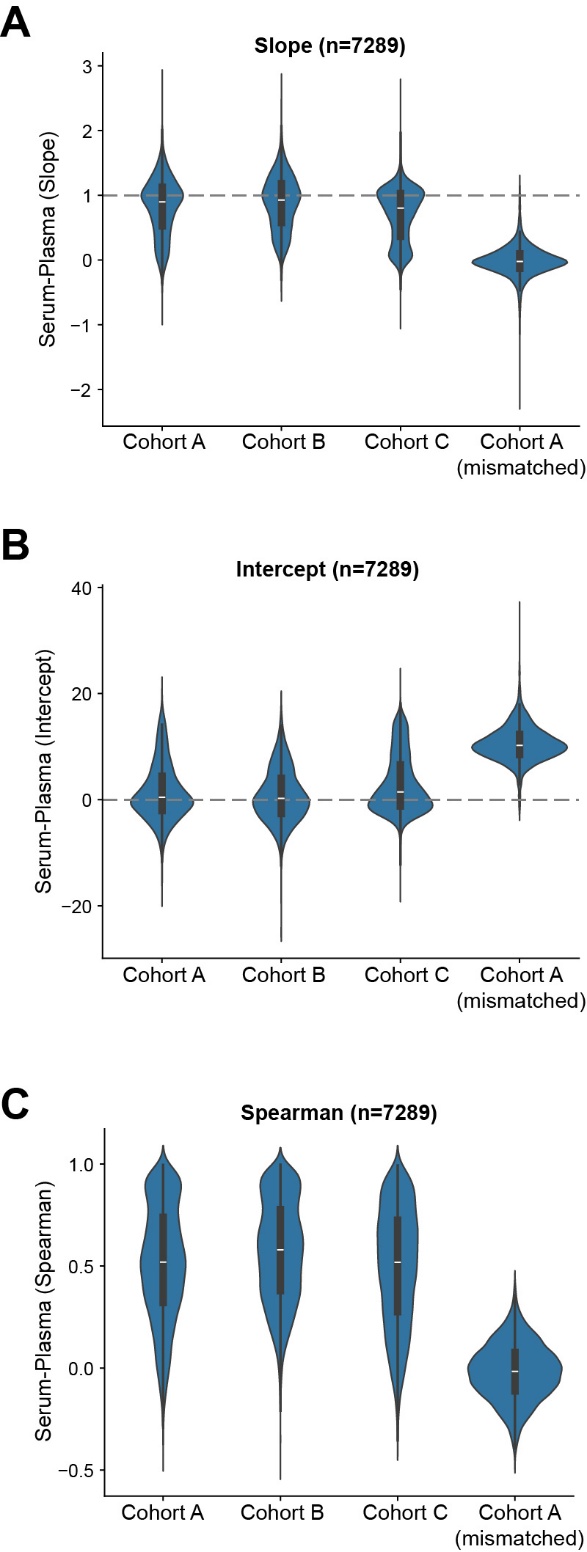


**Supplementary Figure S10: Comparison of linear scaling parameters derived from different cohorts.** Violin plots showing distributions of linear scaling parameters and serum-plasma correlation coefficients based on cohort A, cohort B, cohort C, and mismatched cohort A paired datasets. The analysis was performed for all 7,289 proteins. (A) Slope distributions. The horizontal dashed line denotes slope = 1 (indicating a similar scale for serum and plasma). (B) Intercept distributions. The horizontal dashed line denotes intercept = 0 (indicating the absence of a systematic bias between serum and plasma). (C) Correlation coefficient distributions.


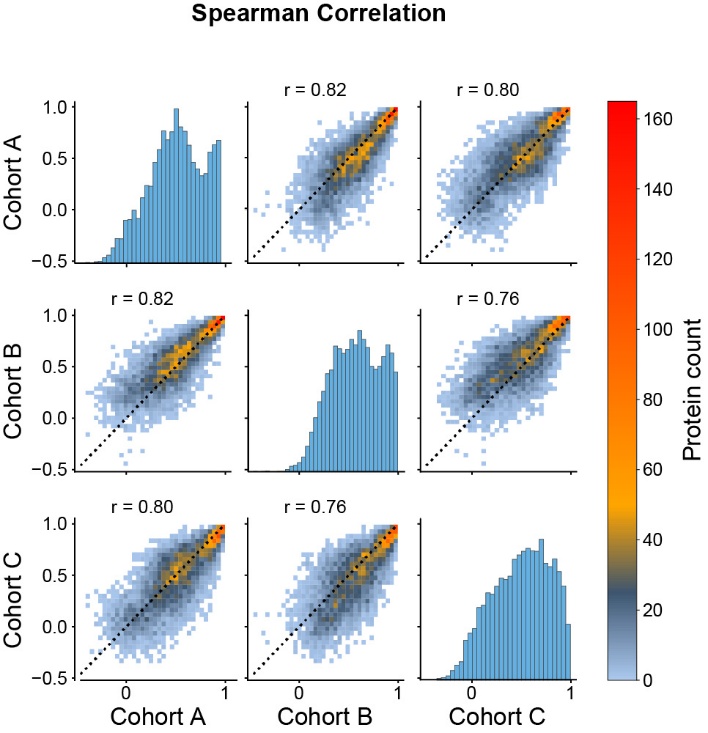


**Supplementary Figure S11: Inter-cohort agreement of serum-plasma protein correlations.** Protein-level Spearman correlation coefficients between serum and plasma protein measurements (n=7,289 proteins) were compared across the three cohorts in a pairwise manner. Diagonal histograms represent the distribution of Spearman correlation coefficients within each cohort. Off-diagonal 2D histograms depict between-cohort value distributions, with Pearson correlation coefficients (r) shown for each comparison. The black dotted line indicates perfect agreement between pairs.

**
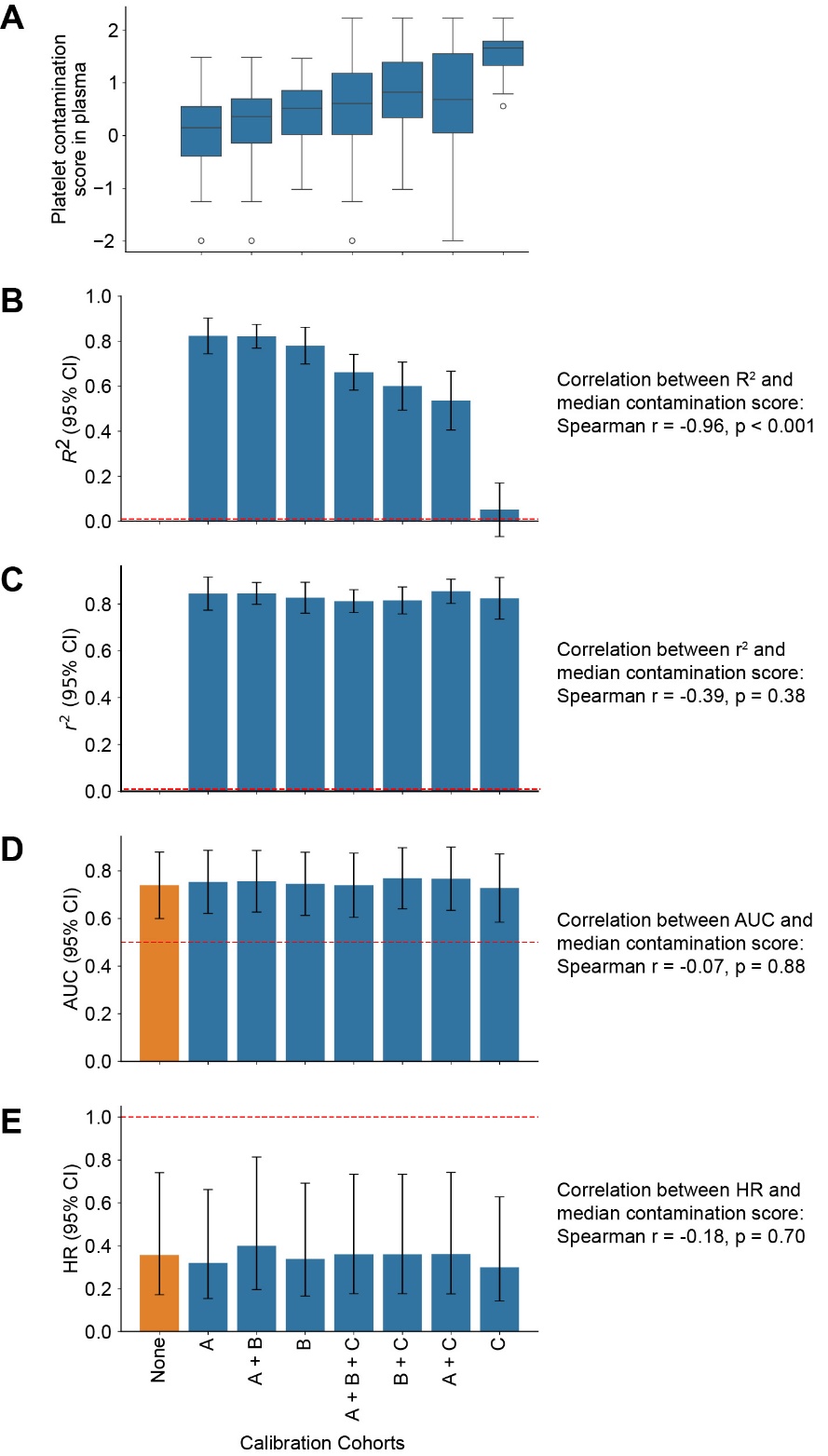
**

**Supplementary Figure S12: Effect of scaling using combined cohorts and all possible cohort combinations.** (A) Platelet contamination scores for plasma samples from combined cohorts, all possible cohort combinations, and individual cohorts. (B-E) Serum proteomic measurements of cohort A were corrected using scaling factors derived from serum-plasma pairs per cohort combination. PROphet performance was assessed using cohort A’s plasma or scaled-serum proteomic data as input. Agreement between plasma-based prediction of clinical benefit (CB) probability versus scaled serum-based prediction of CB probability is shown in (B) and (C), where R^2^ indicates agreement with the identity line (x=y), and r^2^ indicates the agreement with the best-fit line. Area under the curve (AUC) of Receiver Operating Characteristics plots for CB prediction are shown in (D). Hazard Ratios (HR) between PROphet-POSITIVE and PROphet-NEGATIVE groups are shown in (E). Plasma-based (orange bars) and scaled serum-based (blue bars) predictions are shown. Dashed red lines indicate expected values under the null hypothesis. Error bars indicate 95% confidence intervals (CI) calculated using the Olkin-Finn approximation for R² and r², DeLong's method for AUC, and Wald method for HR.


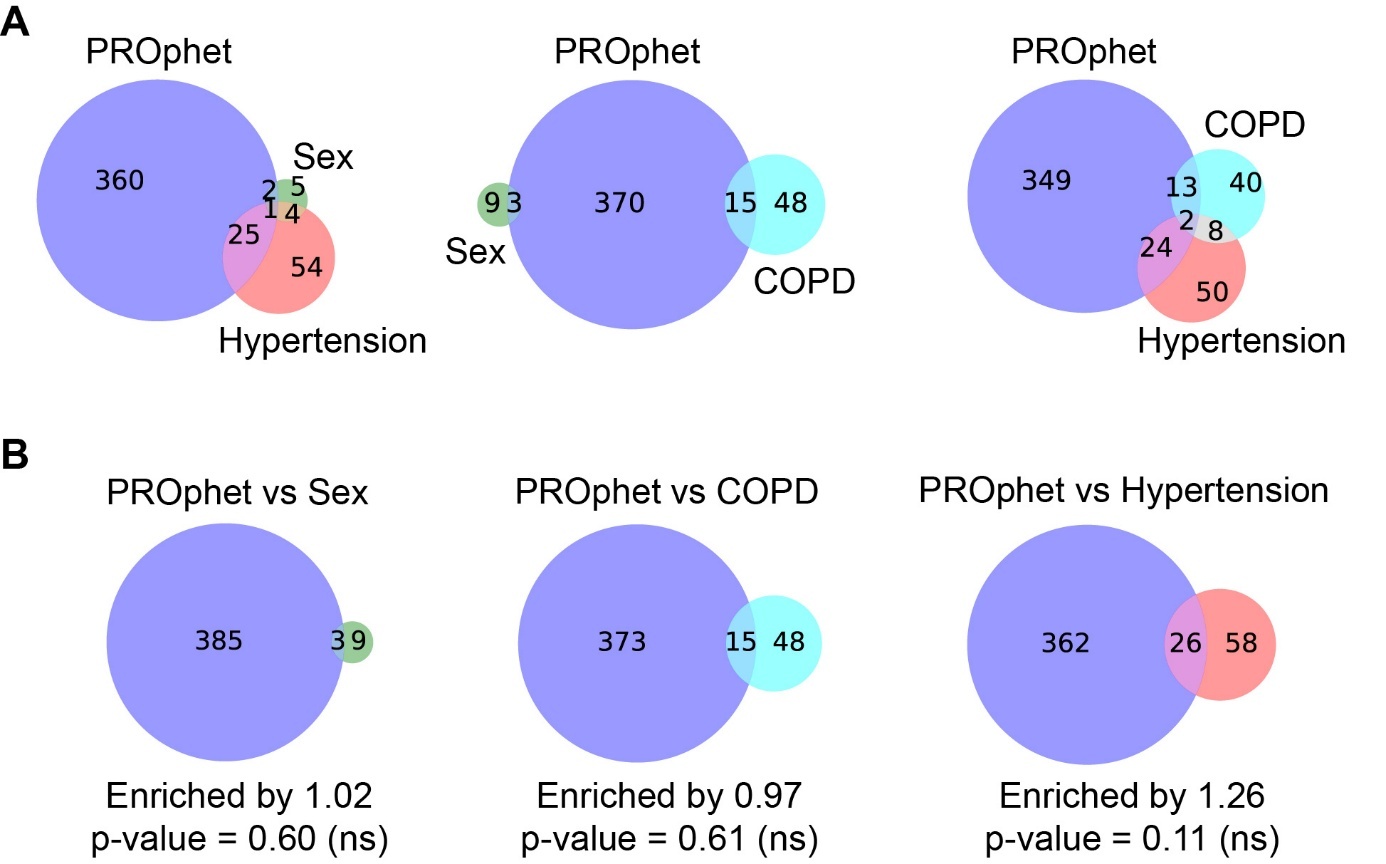


**Supplementary Figure S13: Overlap between biomarkers associated with the PROphet model and three other predictive models.** (A) Three-set Venn diagrams of proteomic biomarkers associated with the PROphet model and models for predicting sex, chronic obstructive pulmonary disease (COPD) and hypertension in patients with non-small cell lung cancer. (B) Two-set Venn diagrams comparing biomarkers of the indicated models. Enrichment factors and p-values show that biomarkers associated with models predicting sex, COPD or hypertension are not significantly enriched for PROphet biomarkers. Background: 1,578 proteins included in model training. ns, not significant.

**
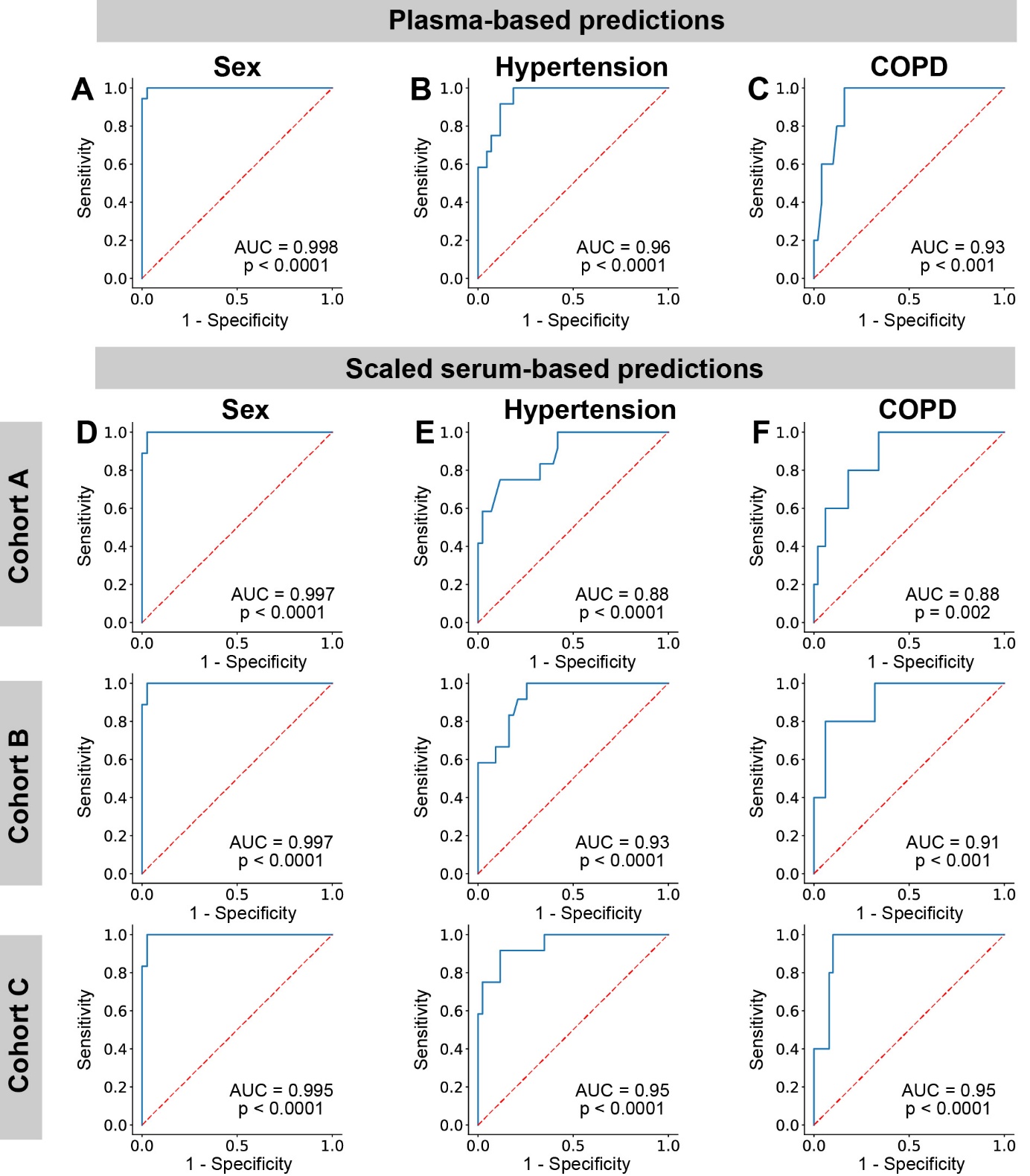
**

**Supplementary Figure S14: Model performance for predicting sex, hypertension and chronic obstructive pulmonary disease.** Models for predicting sex, hypertension and chronic obstructive pulmonary disease (COPD) in patients with non-small cell lung cancer were developed on a set of 228 plasma samples (the same set of samples used for PROphet model development). Predictive performance of each model was evaluated in an independent validation set composed of cohort A samples (n=55) using Receiver Operating Characteristics (ROC) plots. (A-C) Performance of the indicated models with cohort A plasma protein dataset as input. (D-F) Performance of the indicated models with cohort A serum proteomic data corrected with scaling factors derived from cohort A (in cross-validation), cohort B or cohort C, as indicated. AUC, area under the curve.
